# MAIT cell inhibition promotes liver fibrosis regression via macrophage phenotype reprogramming

**DOI:** 10.1038/s41467-023-37453-5

**Published:** 2023-04-01

**Authors:** Morgane Mabire, Pushpa Hegde, Adel Hammoutene, Jinghong Wan, Charles Caër, Rola Al Sayegh, Mathilde Cadoux, Manon Allaire, Emmanuel Weiss, Tristan Thibault-Sogorb, Olivier Lantz, Michèle Goodhardt, Valérie Paradis, Pierre de la Grange, Hélène Gilgenkrantz, Sophie Lotersztajn

**Affiliations:** 1 Université Paris Cité, INSERM, UMR-S1149, Centre de Recherche sur l’Inflammation (CRI), Laboratoire d’Excellence Inflamex, F-75018 Paris, France; 2 Département d’Anesthésie et Réanimation, Hôpital Beaujon, Assistance Publique-Hôpitaux de Paris, 92110 Clichy, France; 3 Institut Curie, INSERM U932, Paris, France; 4 Université Paris Cité, INSERM UMRS 976, Institut de Recherche Saint Louis, F-75010 Paris, France; 5 Département de Pathologie, Hôpital Beaujon, Assistance Publique-Hôpitaux de Paris, 92110 Clichy, France; 6 GenoSplice, Paris, France

**Keywords:** Monocytes and macrophages, RNA sequencing, Liver fibrosis, Innate lymphoid cells

## Abstract

Recent data have shown that liver fibrosis can regress even at later stages of cirrhosis and shifting the immune response from pro-inflammatory towards a resolutive profile is considered as a promising option. The immune regulatory networks that govern the shift of the inflammatory phenotype and thus potential reversal of liver fibrosis are lesser known. Here we show that in precision-cut human liver slices obtained from patients with end-stage fibrosis and in mouse models, inhibiting Mucosal-Associated Invariant T (MAIT) cells using pharmacological or antibody-driven approaches, limits fibrosis progression and even regresses fibrosis, following chronic toxic- or non-alcoholic steatohepatitis (NASH)-induced liver injury. Mechanistic studies, combining RNA sequencing, in vivo functional studies (performed in male mice) and co-culture experiments indicate that disruption of the MAIT cell-monocyte/macrophage interaction results in resolution of fibrosis both by increasing the frequency of restorative Ly6C^lo^ at the expenses of pro-fibrogenic Ly6C^hi^ monocyte-derived macrophages and promoting an autophagic phenotype in both subsets. Thus, our data show that MAIT cell activation and the consequential phenotype shift of liver macrophages are important pathogenic features of liver fibrosis and could be targeted by anti-fibrogenic therapy.

## Abstract (web summary)

Liver cirrhosis is characterised by extensive fibrosis of the liver, and understanding the underpinning immunological processes is important in designing intervention. Here authors show that Mucosal-Associated Invariant T cells are instrumental to controlling the balance between profibrogenic and restorative macrophages and inhibiting their activation might reverse liver fibrosis.

## Introduction

Chronic liver injury exposes to fibrosis and its end-stage cirrhosis is associated with life-threatening complications. Although cirrhosis has long been considered irreversible, data from patients with chronic liver diseases and murine models have clearly established that fibrosis can revert, even at later stages, following the elimination of the underlying cause^1,2^. However, in many cases this goal cannot be achieved, highlighting the urgent unmet need for therapies halting progression, and/or promoting regression of established fibrosis/cirrhosis. Removal or elimination of the causative agent promotes inhibition of the inflammatory and profibrogenic response, together with deactivation and/or disappearance of fibrogenic cells, leading to the resolution of liver fibrosis^3–7^. Among immune cells, different subsets of liver macrophages play a dual role in the control of fibrosis progression and regression^8,9^. In particular, data from human samples and experimental mouse models have shown that during liver injury, recruited monocyte-derived macrophages (MoMac) with high expression of Ly6C secrete pro-inflammatory and profibrogenic cytokines which perpetuate the fibrogenic response^8–10^. In contrast, studies in mouse models have shown that during fibrosis regression, a restorative MoMac population with a Ly6C^lo^ phenotype promotes scar degradation^8,9^. Therefore, controlling the profibrogenic vs restorative macrophage balance is considered an attractive strategy to promote fibrosis regression.

Mucosal-Associated Invariant T (MAIT) cells are non-conventional innate-like T cells at the interface between innate and adaptive immunity^11^. They are restricted by the non-polymorphic MHC-related class 1 (MR1) molecule and express the evolutionary conserved αβ-T cell receptor (TCR), consisting of an invariant alpha chain Vα7.2-Jα33 in humans (Vα19-Jα33 in mice) and a restricted set of TCRβ chains^11,12^. MAIT cells recognize bacterial ligands presented by MR1, that comprise agonist metabolites derived from the synthesis of riboflavin (vitamin B2) biosynthesis pathway, as well as ligands derived from folate metabolism that behave as antagonists^11,13–15^. In particular, 6-Formylpterin (6-FP) and acetyl-6-formylpterin (Ac-6-FP), the resulting products of folic acid photodegradation, can bind to MR1, increasing its stability and its surface expression, but inhibit MAIT cell activation by competing with the activating ligands^16^. The regulatory role of MAIT cells in the pathogenesis of chronic liver diseases has recently emerged^11^. In particular, we and others have shown that MAIT cells are decreased in the blood and in the liver of patients with fibrosis or end-stage cirrhosis^17,18^. However, they accumulate in the fibrotic septa and display an activated phenotype associated with profibrogenic properties, via direct effects on hepatic myofibroblasts, and may promote fibrosis progression, by fostering a local inflammatory reaction^18^. These findings identified MAIT cells as a potential target for the prevention of liver fibrosis progression. In the present study, we combine studies in human precision-cut liver slices (PCLS) and mouse models to investigate whether a MAIT cell inhibition-based strategy may impact liver fibrosis progression and regression. We show that inhibiting MAIT cell activation halts fibrosis progression and promotes fibrosis regression via a change in the profibrogenic vs resolutive macrophage signature and an increase in Ly6C^lo^ restorative MoMac frequency.

## Results

### MAIT cell inhibition reduces inflammatory and fibrogenic genes in human PCLS

**Fig. 1:**
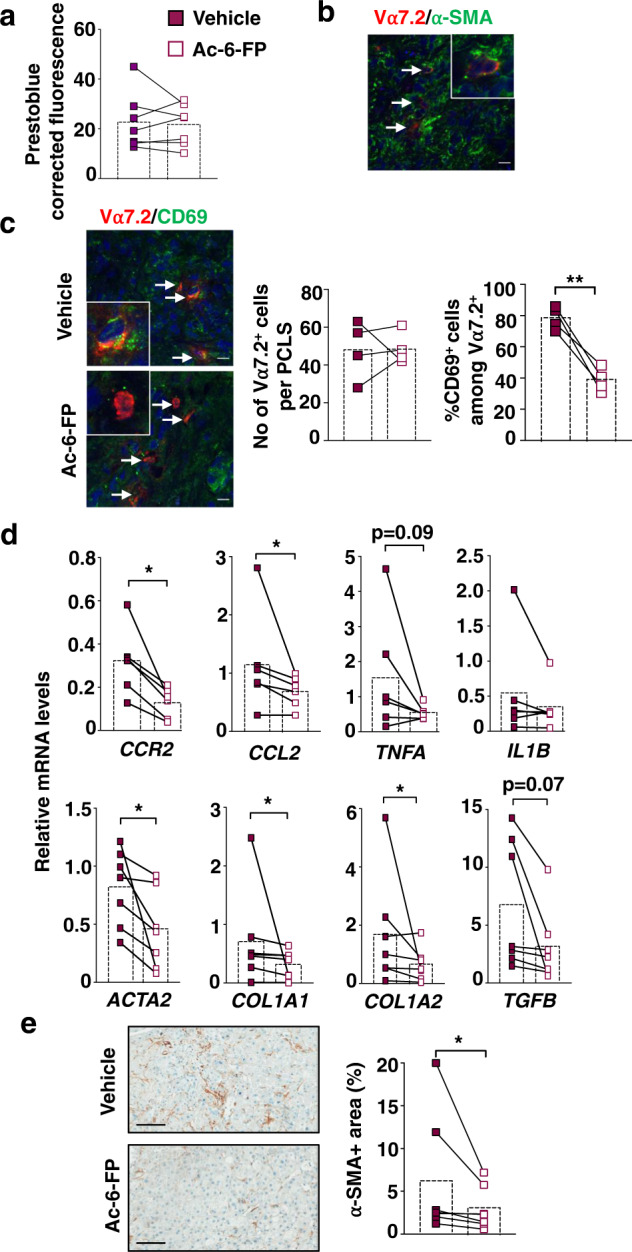
Ex vivo exposure of PCLS from patients with chronic liver injury to the MR1-blocking ligand Ac-6-FP decreases the expression of inflammatory and fibrogenic genes. Human PCLS were incubated with 10 µM Ac-6-FP or its vehicle for 48 h. **a** PCLS viability was assayed using PrestoBlue™ Cell Viability assay by fluorescence. **b** MAIT cell localization was evaluated by immunofluorescent co-staining of Vα7.2 and α-SMA. A representative image of two experiments is shown. **c** Representative images and quantification of Vα7.2+ cell number per PCLS and %CD69 + Vα7.2+ activated cells. Results are expressed as % of CD69^+^Vα7.2^+^/total Vα7.2^+^ cells. Each point is the mean value per PCLS. **d** Expression of inflammatory and fibrogenic genes in PCLS normalized to housekeeping gene PPIA. **e** Representative images of α-SMA immunostaining on liver tissue sections from those cirrhotic patients and respective quantifications of positive areas. **a**, **d**, **e** *n* = 7 patients (see Table 1 Group 1) except for *CCR2*, *TNFA,* and *IL1B* where expression was not detected for one patient. **c** Experiments were performed on four patients (see Table 1 Group 2) using two-tailed paired *t* test (**p = 0.006). **d**, **e** *p < 0.05 by two-tailed Wilcoxon matched-pairs signed rank test. Bars show the mean. Scale bar is 10 µm for **b**, **c** and 100 µm for **e**. Source data are provided as a Source Data file.

We evaluated the consequences of MAIT cell inhibition on liver fibrogenesis in human liver samples, using ex vivo PCLS from patients with fibrosis and end-stage cirrhosis from various etiologies (viral, metabolic and alcoholic, Table 1). PCLS were exposed for 48 h to the non-agonist synthetic folate derivative Acetyl-6-formylpterin (Ac-6-FP), which dampens MR1 stimulation and inhibits MAIT cell activation^16^. There was no impact of Ac-6-FP on the viability of PCLS (Fig. 1a). As previously observed in surgical liver samples from patients with cirrhosis^18^, MAIT cells, identified by their Vα7.2 positivity by immunostaining, were found in proximity to fibrogenic smooth muscle alpha-actin (α-SMA) positive myofibroblasts in PCLS from patients with fibrosis or cirrhosis (Fig. 1b). Exposure of PCLS to Ac-6-FP did not affect the number of Vα7.2+ cells but strongly reduced their activation, as reflected by the decreased number of CD69 + Vα7.2+ cells assessed by immunostaining (Fig. 1c). The MAIT cell antagonist significantly decreased the expression of *CCL2* chemokine and its receptor *CCR2* but did not significantly modify *TNFA* (*p* = 0.09) and *IL1B* expression*s* (Fig. 1d, Table 2). Interestingly, Ac-6-FP caused a significant reduction in the expression of the fibrogenic genes *COL1A1* and *A2*, *ACTA2*, and there was a trend to decrease that of the fibrogenic cytokine *TGFB* (*p* = 0.07) (Fig. 1d). These data were further confirmed by a decrease in the number of α-SMA+ cells in response to Ac-6-FP (Fig. 1e). These findings indicate that inhibiting MAIT cell activation decreases inflammatory and fibrogenic gene expression in human liver samples.

**Table 1:** Patients’ characteristics

Patient	Age	Gender	Underlying liver disease	Etiology of fibrosis	Fibrosis score
Group 1					
1	73	M	HBV related HCC	HBV	F3
2	70	M	HCV related HCC	HCV	F4
3	49	M	Alcohol and metabolic cirrhosis (explanted liver)	Alcohol and NAFLD	F4
4	65	M	Alcohol-related HCC (explanted liver)	Alcohol	F4
5	33	M	HBV related HCC	HBV	F4
6	61	M	HCV related HCC	HCV	F4
7	65	F	Intrahepatic cholangiocarcinoma	NAFLD	F4
Group 2					
8	51	M	Alcohol-related cirrhosis (explanted liver)	Alcohol	F4
9	68	F	Metabolic cirrhosis (explanted liver)	NAFLD	F4
10	69	M	Alcohol and metabolic-related HCC	Alcohol and NAFLD	F3/4
11	54	F	Intrahepatic cholangiocarcinoma	NAFLD	F2

*HBV* hepatitis B virus, *HCC* hepatocellular carcinoma, *HCV* hepatitis C virus, *NAFLD* non-alcoholic fatty liver disease.

Non-tumoral parenchyma was used for PCLS.

**Table 2:** TaqMan® probes used for RT-qPCR

GENE	TAQMAN PROBE ID
*ACTA2*	Hs00426835_g1
*CCL2*	Hs00234140_m1
*COL1A1*	Hs00164004_m1
*COL1A2*	Hs01028956_m1
*IL1B*	Hs01555410_m1
*PPIA*	Hs04194521_s1
*TGFB1*	Hs00998133_m1
*TNFA*	Hs00174128_m1
*CCR2*	Hs00356601_m1

TaqMan® probes were supplied by Applied Biosystems.

### MAIT cell inhibition blocks liver fibrosis progression and promotes regression

**Fig. 2:**
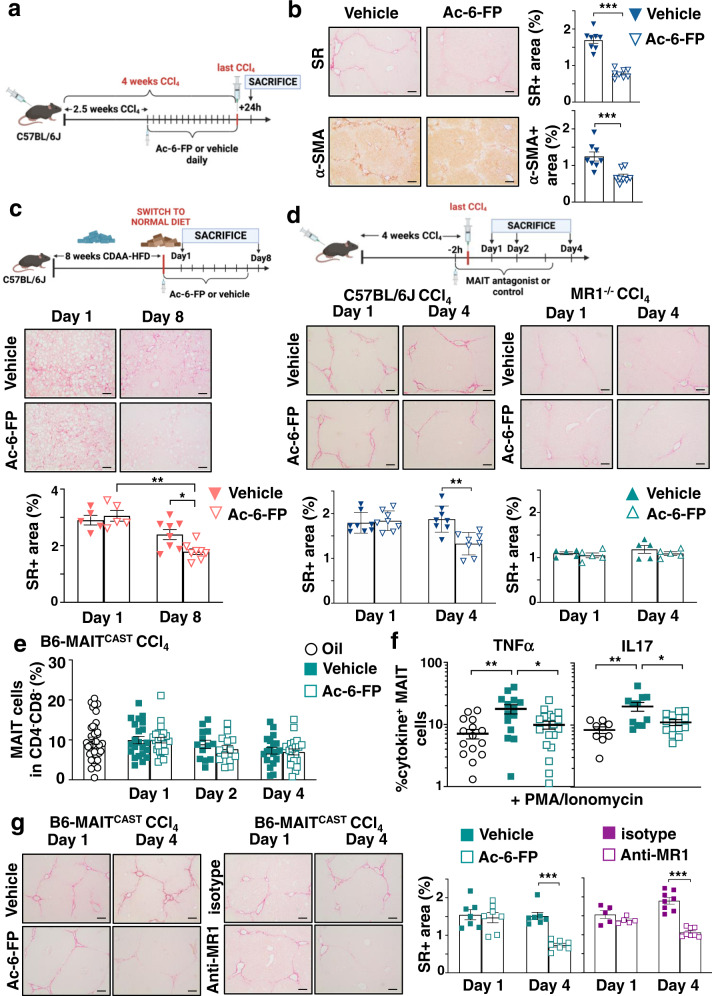
Inhibition of MAIT cell activation blocks liver fibrosis progression and promotes fibrosis regression. **a** Timeline of the CCl_4_-induced fibrosis progression protocol in C57BL/6 J mice. **b** Representative images and quantification of Sirius red (SR; ***p = 0.0002) and α-SMA-positive areas (***p = 0.0006) in liver tissue sections (*n* = 8 mice/group). **c** Timeline of the CDAA-HFD-induced fibrosis regression protocol in C57BL/6 J mice. Representative images and quantification of Sirius red areas in liver tissue sections at day 1 (*n* = 5 mice/group) and day 8 (*n* = 8 mice/group). *p = 0.02; **p = 0.0016. **d** Timeline of the CCl_4_-induced fibrosis regression protocol in C57BL/6 J and MR1^−/−^ mice. Representative images and quantification of Sirius red areas in liver tissue sections from C57BL/6 J (*n* = 8 mice/group) and MR1^−/−^ mice (*n* = 5 mice/group) at days 1 and 4. **p = 0.002. **e** Flow cytometry analysis of CD4^−^CD8^−^GFP^+^ MAIT cell frequency in CCl_4_-exposed B6-MAIT^CAST^ mice injected with Ac-6-FP or vehicle (pooled data from three experiments *n* = 22 mice in vehicle group and 25 mice in Ac-6-FP group at day 1; *n* = 13 mice/group at day 2; *n* = 19 mice for vehicle and 21 mice for Ac-6-FP at day 4). Oil-injected mice served as control (pooled data from 7 experiments *n* = 36 mice). **f** Intracellular staining of CD4^−^CD8^−^GFP^+^ MAIT cells for TNFα and IL17 after 4 h intrahepatic leukocyte stimulation with PMA/ionomycin and Brefeldin A. Pooled data from 3 experiments for TNFα at day 1 (*n* = 16 mice for oil, *n* = 14 mice for vehicle and *n* = 18 mice for Ac-6-FP; *p = 0.04, **p = 0.003), and from 2 experiments for IL17 (*n* = 9 for oil, *n* = 11 for vehicle and *n* = 12 for Ac-6-FP; *p = 0.03, **p = 0.004). **g** Representative images and quantification of Sirius red areas in liver tissue sections from B6-MAIT^CAST^ congenic mice daily injected either with Ac-6-FP or vehicle (*n* = 7 mice/group; ***p = 0.0006), anti-MR1 or isotype (*n* = 5 mice/group at day 1, *n* = 8 at day 4; ***p = 0.0002). **a**, **c**, **d** Timelines of injections were created with BioRender® software. Representative images were taken at ×10 magnification. Scale bar is 100 µm. Data are mean ± S.D. Statistical analysis was performed by two-tailed Mann–Whitney test. SR sirius red, CDAA-HFD choline-deficient l-amino-acid defined high-fat diet, PMA phorbol 12-meristate 13-acetate. Source data are provided as a Source Data file.

We next evaluated whether blocking MAIT cells in a therapeutic setting (i.e., administration of Ac-6-FP at the stage of fibrosis, while continuing the injury) may prevent fibrosis progression. To that aim, male mice were administered CCl_4_ for 4 weeks, with daily injection of Ac-6-FP starting from 2.5 weeks, at a time point where mild fibrosis is already observed^19^ (Fig. 2a). We found that blocking MAIT cell activation limits fibrosis progression, as shown by reduced Sirius red staining and decreased number of α-SMA+ cells in mice administered Ac-6-FP as compared to vehicle-injected animals (Fig. 2b). These data demonstrate the efficacy of therapeutic intervention targeting MAIT cells for liver fibrosis.

We also evaluated whether blocking MAIT cell activation impacts fibrosis regression in mice. We used two mouse models of fibrosis reversibility, which consist either in the switch to normal diet from Choline-Deficient, L-Amino-Acid defined High Fat Diet (CDAA-HFD), a model of non-alcoholic steatohepatitis (NASH)-induced fibrosis, or the cessation of CCl_4_ administration in mice with established CCl_4_-induced fibrosis^8,20,21^. In the first model, male C57BL/6 J mice were fed with CDAA-HFD for 8 weeks, and switched to a normal diet together with injection with Ac-6-FP for 1 day, or daily for 8 days after switching (Fig. 2c). Switching to a normal diet for 8 days led to steatosis reversal in vehicle-exposed mice, as shown by the decrease in the steatosis area, that was not affected by administration of Ac-6-FP (Fig. S1a). In contrast, switching to a normal diet did not affect the extent of collagen accumulation and fibrogenic cell density in vehicle-injected mice as compared to day 1, but Ac-6-FP promoted fibrosis regression, as shown by the reduction of Sirius red (Fig. 2c) and α-SMA (Fig. S1b) staining areas, compared to vehicle at day 8. In the second model, male C57BL/6 J mice were chronically i.p administered CCl_4_ for 4 weeks, and injected Ac-6-FP for 1 day, or daily for 4 days after the last CCl_4_ injection (Fig. 2d). As previously described^20^, accumulation of collagen and the density of α-SMA-expressing fibrogenic cells remained elevated 4 days after the last CCl_4_ injection, in C57BL/6 J mice injected with vehicle. In contrast, mice daily administered Ac-6-FP showed fibrosis regression, as reflected by reduced Sirius red (Fig. 2d) and α-SMA staining areas (Fig. S1c). Importantly, Ac-6-FP did not modify Sirius red staining when administered to male MR1^−/−^ mice, which are deficient in MAIT cells (Fig. 2d). These data, obtained in two different settings, i.e., a NASH and a toxic model of liver fibrosis, demonstrate that inhibiting MAIT cell activation promotes fibrosis regression.

The efficacy and specificity of Ac-6-FP were further evaluated in B6-MAIT^CAST^ mice^22^. These mice are enriched in MAIT cells and constitute a relevant tool for functional evaluation of MAIT cells, since they have been bred with a transgenic RORγtGFP reporter strain, and express GFP in RORγT-expressing cells, including MAIT cells^22^. The frequency of liver MAIT cells, identified as CD45+ CD19−CD11b−TCRβ+ CD4−CD8−RORγtGFP+ (Fig. S2) was not modified upon exposure to CCl_4_ and was not affected by Ac-6-FP (Fig. 2e). Similar findings were obtained when identifying MAIT cells with MR1 tetramers loaded with 5-OP-RU (CD45+ CD19-CD11b-TCRβ+ CD4−CD8-MR1tet+, Fig. S3a). The compound did not alter the frequency of total TCRβ+, CD4+, CD8+, CD4^−^CD8^−^, NK1.1+, γδT, or NKT cells, either in B6-MAIT^CAST^ mice or C57BL/6 J mice (Fig. S3b, c). We previously observed that in patients with end-stage liver disease, MAIT cells display an activated phenotype^18^. In keeping, in mice chronically exposed to CCl_4_, liver MAIT cells also exhibited an activated phenotype characterized by an increased frequency of TNFα+ and IL17 + MAIT cells, as compared to that of oil-injected animals (Fig. 2f). The MR1-blocking ligand Ac-6-FP decreased MAIT cell activation, since it abolished the increase in TNFα+ or IL17+ liver MAIT cell frequency in CCl_4_-injected mice (Fig. 2f). As observed in C57BL/6 J mice, the blocking ligand also promoted fibrosis regression in B6-MAIT^CAST^ mice after 4 days (Fig. 2g).

Beneficial impact of a MR1-blocking strategy was further confirmed with a monoclonal neutralizing antibody to MR1. Injection of the MR1 antibody to mice with established fibrosis for 4 days after the last CCl_4_ injection decreased MAIT cell activation, as reflected by reduced frequency of CD69+ MAIT cells (Fig. S3d) while it did not affect MAIT cell frequency (Fig. S3e). Mice injected with the MR1 antibody showed decreased Sirius red staining compared to mice injected with isotype (Fig. 2g). Taken together, these data demonstrate through two different, pharmacological and antibody-driven, approaches the beneficial impact of MAIT cell inhibition on fibrosis regression.

### Inhibiting MAIT cells alters the frequency and phenotype of MoMac

**Fig. 3:**
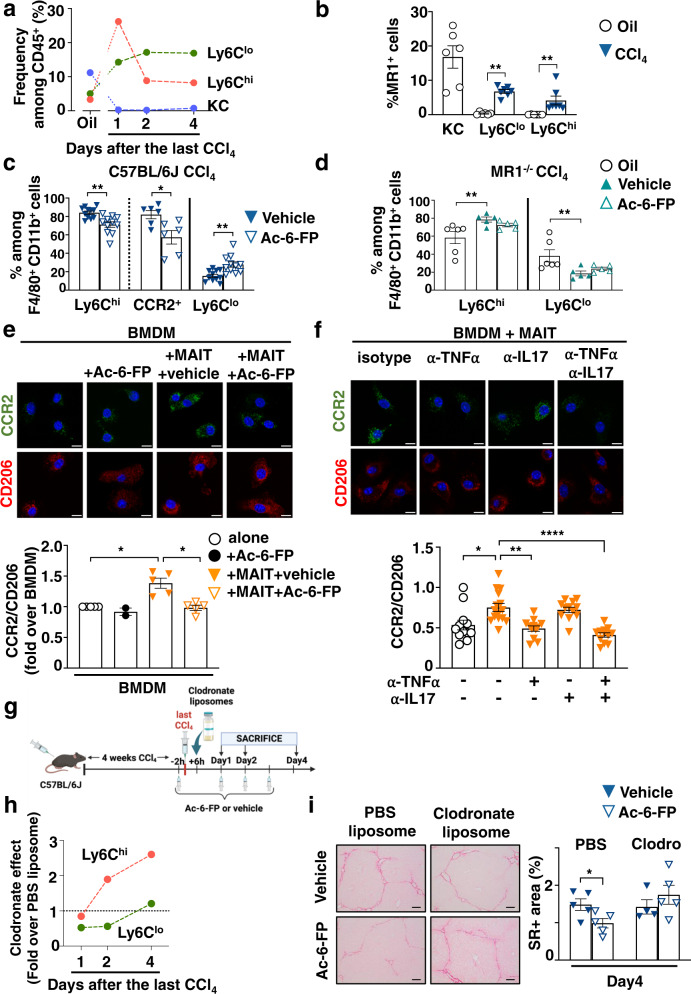
Ac-6-FP promotes fibrosis resolution via an impact on the MAIT macrophage dialog. **a**–**d** Mice were injected CCl_4_ or oil for 4 weeks along the protocol described in Fig. 2d and frequencies of resident Kupffer cells (KC) and MoMac analyzed after cessation of injury. **a** Frequency of intrahepatic KC, Ly6C^lo^ and Ly6C^hi^ MoMac in C57BL/6 J. Each point is the mean of five mice except for oil where *n* = 13 mice. **b** Frequencies of MR1^+^ cells among KC, Ly6C^lo^ and Ly6C^hi^ MoMac at day 1 after CCl_4_ cessation (*n* = 7 mice/group) vs oil (*n* = 6 mice/group). **p = 0.001. **c** Frequencies of Ly6C^hi^, Ly6C^lo^ (pooled data from two experiments, *n* = 11 mice/group), and CCR2^+^ (*n* = 6 mice/group) MoMac from C57BL/6 J mice at day 1 after the last CCl_4_ injection. *p = 0.01, **p = 0.002. **d** Frequencies of Ly6C^hi^ and Ly6C^lo^ from MR1^−/−^ mice exposed to Ac-6-FP or vehicle, at day 1 after CCl_4_ cessation (*n* = 5 mice/group) compared to oil (*n* = 6). **p = 0.009. **e**, **f** Representative images of immunofluorescent staining of CD206 and CCR2 and quantification of the CCR2/CD206 mean intensity ratio in BMDM alone or co-cultured with MAIT cells for 48 h in the presence of **e** Ac-6-FP or vehicle (*n* = 5 experiments except for BMDM + Ac-6-FP where *n* = 2; each point represent the mean per experiment; *p = 0.02 for BMDM + MAIT vs BMDM alone, and *p = 0.04 for BMDM + MAIT + Ac-6-FP vs BMDM + MAIT + vehicle), or **f** antibodies to TNFα and/or IL17 or control isotype (typical representative of two experiments; each point is one field; *n* = 12 fields/group except for BMDM + MAIT + isotype where *n* = 15 fields). *p = 0.02; **p = 0.002; ****p < 0.0001. Scale bar is 10 µm. **g** Timeline of CCl_4_, Ac-6-FP and clodronate or PBS liposome administration in C57BL/6 J mice created with BioRender® software. **h** Clodronate vs PBS liposome fold change of Ly6C^hi^ and Ly6C^lo^ MoMac frequencies at day 1, 2, and 4 after CCl_4_ cessation. Each point represents the mean value of 5 mice for day 1 and day 2, and 4 mice for day 4. **i** Representative images and quantification of Sirius red areas (typical representative of *n* = 4 experiments) of liver tissue sections from mice treated with Ac-6-FP or vehicle (*n* = 5 mice/group except for vehicle clodronate group where *n* = 4 mice). Sample were collected 4 days after the last CCl_4_ injection. Scale bar is 100 µm. *p = 0.05. Data are mean ± S.D. Statistical analysis was performed by (**b**–**d, i**) two-tailed Mann–Whitney test or (**e**, **f**) Kruskal–Wallis followed by Dunn’s multiple comparisons post-test. KC Kupffer cells, BMDM bone marrow-derived macrophages, clodro clodronate-encapsulated liposomes, PBS phosphate-buffered saline-encapsulated liposomes. Source data are provided as a Source Data file.

MoMac play a dual role in fibrosis progression and regression, with CCR2-dependent recruitment of Ly6C^hi^ inflammatory monocytes to the liver during fibrosis progression, and a shift to Ly6C^lo^ restorative monocyte-derived macrophages (MoMac) during resolution^8,21,23^. We, therefore, evaluated their contribution to the acceleration of fibrosis regression induced by MAIT cell inhibition. Time course flow cytometry analysis of monocyte/macrophage populations during fibrosis regression showed that, among the total CD45+, Kupffer cells (F4/80+TIM4+) were the main macrophage population in the liver of control oil-injected mice (Fig. 3a). However, the proportion of Kupffer cells was strongly decreased by CCl_4_ at day 1 and up to 4 days after cessation of the injury. In contrast, and as previously reported^8^ the proportion of profibrogenic Ly6C^hi^ MoMac population strongly increased one day after cessation of injury but decreased thereafter; the restorative Ly6C^lo^ MoMac population emerged one day after cessation of the injury and was sustained for up to 4 days (Fig. 3a, Fig. S4a). Interestingly, flow cytometry analysis showed that Kupffer cells were the only population expressing MR1 among the monocyte/macrophages in control oil-injected mice, with marginal expression in Ly6C^lo^ and Ly6C^hi^ MoMac. In CCl_4-_injected animals, where Kupffer cells are lost, the Ly6C^lo^ and to a lesser extent Ly6C^hi^ MoMac populations convert into MR1-expressing cells, as evidenced by up-regulation of MR1 expression at the cell surface (Fig. 3b).

Flow cytometry analysis of hepatic leukocytes showed that administration of Ac-6-FP resulted in a decrease in the frequency of the Ly6C^hi^ and CCR2^+^ MoMac, and a concordant increase in the Ly6C^lo^ MoMac (Fig. 3c, Fig. S4a–c). Strikingly, the frequency of Ly6C^hi^ and Ly6C^lo^ MoMac was not affected by Ac-6-FP in MAIT cell-deficient MR1^−/−^ mice (Fig. 3d). Taken together, these data demonstrate that blocking MAIT cell activation results in a decrease in the recruitment of Ly6C^hi^ /CCR2^+^ MoMac and the emergence of a Ly6C^lo^ MoMac phenotype.

We next investigated whether MAIT cells have a direct impact on macrophage phenotype in co-culture experiments. Naive bone marrow-derived macrophages (BMDM) were co-cultured either alone, or with anti-CD3/CD28-activated MAIT cells exposed to Ac-6-FP or vehicle (FACS gating strategy of MAIT cells Fig. S5). In the presence of activated MAIT cells, BMDM showed an increase in the CCR2/CD206 ratio, reflecting a shift toward a pro-inflammatory macrophage phenotype, while no increase was observed in the presence of Ac-6-FP (Fig. 3e); Ac-6-FP had no direct effect when added alone to BMDM (Fig. 3e). In addition to MR1 requirement, neutralizing antibodies to TNFα also blunted the increase in the CCR2/CD206 ratio, whereas blocking IL17 had no effect (Fig. 3f).

We next examined the consequences of i.v clodronate administration on Ac-6-FP-induced fibrosis regression. Liposome-encapsulated clodronate or Phosphate Buffer Saline (PBS) counterparts were administered to mice with established fibrosis following 4 weeks of CCl_4_ administration, and Ac-6-FP was daily administered for 1, 2, or 4 days following cessation of CCl_4_ exposure (Fig. 3g). Compared to the liposome-encapsulated PBS control group, clodronate treatment depleted half of intrahepatic Ly6C^lo^ MoMac at day 1 and 2, which was restored at day 4. In contrast, clodronate did not deplete Ly6C^hi^ at day 1, in keeping with their low phagocytic capacity^8^, and a compensatory increase was observed from day 2 (Fig. 3h). MAIT cell inhibition by Ac-6-FP did not affect the extent of MoMac depletion (Fig. S6). Finally, whereas Ac-6-FP promoted fibrosis regression in mice exposed to control liposomes, this effect was totally abolished upon depletion with liposome-encapsulated clodronate (Fig. 3i).

Altogether, these data highlight that acceleration of fibrosis regression by Ac-6-FP is supported by a MAIT-monocyte/macrophage dialog, that results in a shift from a pro-inflammatory Ly6C^hi^ toward a restorative Ly6C^lo^ MoMac phenotype.

### Inhibiting MAIT cell activation alters the MoMac signature

**Fig. 4:**
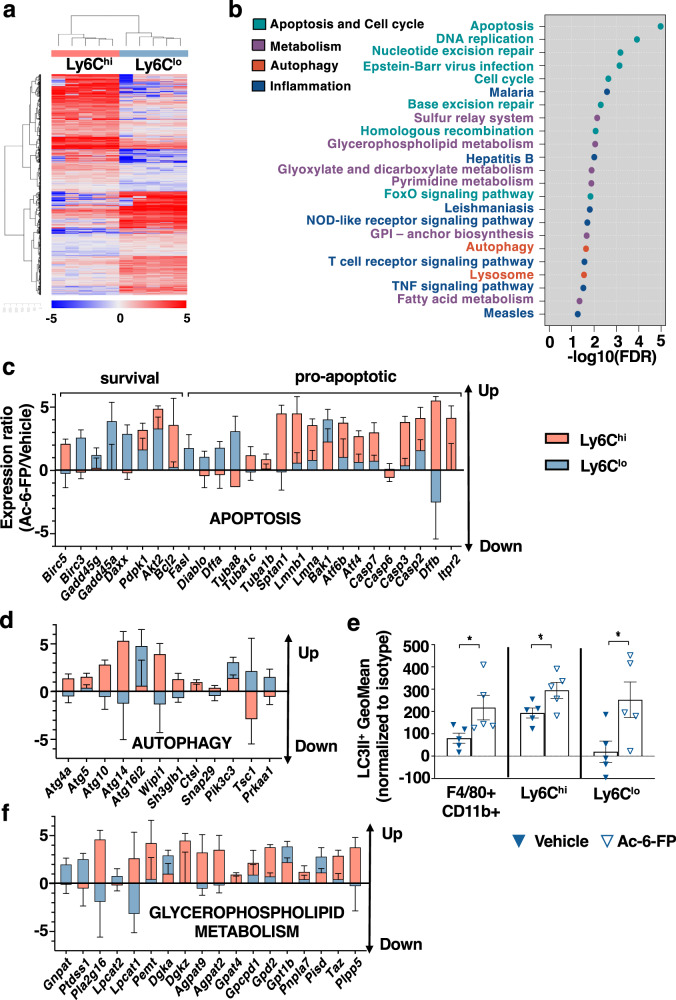
Blocking MAIT cell activation impacts on the Ly6C^hi^ vs Ly6C^lo^ macrophage signature. **a** Heatmap of the 2687 genes with a significant (*p* ≤ 0.05, paired *t* test) difference in the ratio of Ly6C^hi^ Ac-6-FP/vehicle and Ly6C^lo^ Ac-6-FP/vehicle and a fold change ≥ 1.5. Clustering used Euclidean distance and Ward.D2 agglomeration method. **b** Top KEGG pathways from the enrichment analysis based on the 2687 genes with a significant difference in the ratio of Ly6C^hi^ Ac-6-FP/vehicle vs Ly6C^lo^ Ac-6-FP/vehicle. KEGG pathways are ordered by −log10(FDR). **c** Ac-6-FP/mean vehicle ratios in Ly6C^hi^ (red) and Ly6C^lo^ (blue) for selected genes from Apoptosis (mmu04210) and **d** Autophagy (mmu04140) KEGG pathways. **e** Geometric Mean of LC3II-positive cells in total macrophages (F4/80 + CD11b + ; *p = 0.03) and Ly6C^hi^ and Ly6C^lo^ (*p = 0.05) macrophages sorted from C57BL/6 J mice either injected Ac-6-FP or its vehicle. Statistical analyses were performed using two-tailed Mann–Whitney test. **f** Ac-6-FP/mean vehicle ratios in Ly6C^hi^ (red) and Ly6C^lo^ (blue) for selected genes from Glycerophospholipid metabolism (mmu00564). **c**–**f** Experiments were performed on *n* = 5 mice/group. Data are presented as mean values ± S.E.M. Up upregulated genes, Down downregulated genes. Source data are provided as a Source Data file.

In order to determine whether Ac-6-FP-induced inactivation of MAIT cells also impacts on MoMac signature, we performed RNA sequencing analysis on sorted Ly6C^hi^ and Ly6C^lo^ MoMac isolated from the liver of CCl_4_-injected mice exposed to Ac-6-FP or vehicle. Impact of Ac-6-FP on both populations was evaluated by comparing genes differentially regulated between Ac-6-FP in Ly6C^hi^ and Ly6C^lo^ MoMac. A total of 2687 genes were differentially regulated by Ac-6-FP in Ly6C^hi^ vs Ly6C^lo^ MoMac with 1428 genes upregulated and 1259 downregulated (*p* ≤ 0.05 and fold change ≥ 1.5) (Fig. 4a). Among the 89 deregulated KEGG pathways, the first 25 could be classified into “apoptosis/cell cycle”, “metabolism”, “autophagy” and “inflammation” (Fig. 4b).

Apoptosis was far the most significant pathway deregulated by Ac-6-FP in Ly6C^hi^ vs Ly6C^lo^ MoMac. Indeed, Ac-6-FP mainly upregulated apoptotic genes in Ly6C^hi^ MoMac such as members of the caspase family (*Casp 2, 3,* and *7*), but only marginally in Ly6C^lo^ liver MoMac. In contrast, Ac-6-FP enhanced the expression of some anti-apoptotic genes in resolutive Ly6C^lo^ macrophages (Fig. 4c), including A*kt2, Pdpk1* or *Birc3*. Interestingly, the autophagy pathway was also upregulated by Ac-6-FP, with the induction of a set of non-overlapping autophagic genes in Ly6C^hi^ vs Ly6C^lo^ MoMac (Fig. 4d). In particular, genes of the ATG family, such as *Atg4a, Atg5, Atg10, and Atg14*, as well as *Wipi1 or Ctsl* were upregulated by Ac-6-FP in Ly6C^hi^, whereas *Pik3c3, Tsc1 or Prkaa1* were induced in Ly6C^lo^ MoMac. Functional FACS analysis of the autophagy marker LC3II further showed an increase in the frequency of both LC3II + Ly6C^hi^ and Ly6C^lo^ MoMac isolated from the liver of CCl_4_-injected mice exposed to Ac-6-FP (Fig. 4e). Ac-6-FP also deregulated several metabolic pathways, among which one of the most significant pathways was the glycerophospholipid metabolic pathway, converging to phosphatidic acid and phosphatidylcholine synthesis, with 12 genes out of 18 mainly upregulated in Ly6C^hi^ MoMac (Fig. 4f), including *Taz, Gpat4, Agpat2, Agpat9, Dgka,* and *Dgkz, Lpcat1,* *and* *Pemt*. Interestingly, certain lipids including phosphatidic acid are required in the autophagy process during autophagosome formation and fusion with lysosomes^24^. Intriguingly, the impact of Ac-6-FP on the inflammatory signature of macrophages was minor (Fig. S7). Indeed, deregulated pathways (“Malaria”, “Hepatitis B”, “Leishmaniasis”, “NOD-like receptor signaling pathway”, “TNF signaling pathway” and “Measles”) were all related to inflammatory signaling but mainly included genes of the KEGG “apoptosis” or “autophagy” pathways (*Atg5, Bcl2*, *Birc3, Akt2*… Fig. 4c, d). In addition, some genes were not restricted to inflammatory signaling but also related to survival/apoptosis and autophagy, such as *Mapk13, Mapk 14, Tbk1*, *Nfkbia,* and *Nfkbib*. Finally, variations in both pro (*Myd88, Aim2, Il18, Il1b, Ptgs2)* and anti-inflammatory/fibrogenic genes (*Ifng, Ticam1, Tbk1and Mmp9)* were observed in one or both Ly6C^hi^ and Ly6C^lo^ MoMac (Supplementary data 1).

Taken together, these data demonstrate that inhibition of MAIT cell activation by Ac-6-FP shifts Ly6C^hi^ MoMac phenotype toward a profile characterized by enhanced apoptotic and autophagic features and induction of the glycerophospholipid metabolic pathway. The MAIT cell inhibitory ligand also promotes a shift toward resolutive Ly6C^lo^ MoMac with enhanced survival and autophagic features.

## Discussion

Despite major advances in the understanding of the mechanisms underlying the resolution of liver fibrosis and its end-stage cirrhosis, pharmacological therapies are still lacking in humans. Because specific immune cell subsets drive fibrosis progression and regression, recent studies have underscored that anti-inflammatory approaches may serve as the basis for promoting fibrosis resolution. Combining human data in ex vivo precision-cut liver slices with mouse models and cell culture experiments, the present study demonstrates that inhibiting MAIT cell activation by MR1 inhibitory ligands constitutes an interesting strategy to limit liver fibrosis progression and promote fibrosis resolution. Mechanistic studies show that fibrosis resolution initiated by inhibition of MAIT cell activation triggers a change in the profibrogenic vs resolutive macrophage signature and a decrease in their frequency, in favor of Ly6C^lo^ resolutive MoMac phenotype.

The control of inflammatory signals originating from innate, innate-like, and adaptive immunity is considered an interesting option for promoting fibrosis regression^3,4,6^. As such, the capacity of activated MAIT cells to produce profibrogenic cytokines such as IL17 and TNFα makes these immune cells an attractive target for liver fibrosis resolution. As we previously observed in human liver samples^18^, mouse models of chronic liver injury demonstrated that liver MAIT cells were activated at the onset of fibrosis and adopted an IL17/TNFα phenotype, therefore validating their use to test the potential of MAIT cell inhibition-based antifibrogenic strategy. Identification of interventional treatments modulating MAIT cell frequency and/or phenotype is a major challenge. As of now, proposed therapies to block MAIT cell activation favor the blockade of MR1 presentation by MR1 inhibitory ligands such as Ac-6-FP or MR1-blocking antibodies, rather than directly targeting MAIT cells, given their important role in the homeostasis of mucosal tissues against invading pathogens^25^. These MR1 inhibitory ligands have shown their efficacy in preclinical models, with antitumoral effects^26^, beneficial effects on bacterial lung infection^27^ and improved insulin sensitivity and glucose tolerance in obese mice^28^. Using this strategy, we demonstrate that administration of Ac-6-FP following initiation of the CCl_4_-induced injury slows down fibrosis progression, demonstrating the efficacy of targeting MAIT cells in a therapeutic setting. Importantly, we also unravel that acceleration of liver fibrosis regression is promoted by blocking MR1 presentation by either Ac-6-FP or an MR1 antibody. We could extend our findings to a mouse model of NASH-induced fibrosis, in which Ac-6-FP also accelerated fibrosis regression when switching to a normal diet. Interestingly, the antifibrogenic efficacy of Ac-6-FP was also demonstrated in pilot studies using human precision-cut liver slices, which preserve the complex liver architecture and retain cell-cell and cell-matrix contacts. Indeed, exposure of PCLS from patients with fibrosis or end-stage cirrhosis to Ac-6-FP resulted in a decrease in MAIT cell activation and reduced the expression of fibrogenic genes, including *COL1A1* and *COL1A2*, *ACTA2,* and *TGFB1*, together with the number of fibrogenic cells. Interestingly, Ac-6-FP also caused a reduction in the expression of the pro-inflammatory/fibrogenic chemokine *CCL2* and of its receptor *CCR2*, a receptor expressed both by hepatic stellate cells and pro-inflammatory macrophages^29^. The nature of the bacterial-derived MAIT ligand presented by MR1 remains to be characterized but is most likely a gut-derived bacterial component that flows to the liver^13,14^, as a consequence of the characteristic increase in gut permeability, intestinal bacteria overgrowth and dysbiosis observed in patients and animal models during chronic liver injury^30^. Taken together, our data in mice and human samples reveal MAIT cells as one major actor that promotes fibrosis progression and limits resolution during chronic liver injury and validate the use of a MAIT cell-based inhibitory antifibrogenic approach.

Another major finding relates to the demonstration that an MR1-blocking strategy impacts on the MAIT macrophage crosstalk to drive fibrosis regression. Previous studies in mice have undoubtedly established the crucial role of monocytes and macrophages in fibrosis resolution, which is orchestrated by restorative Ly6C^lo^ MoMac at the expense of scar-associated Ly6C^hi^ MoMac^8,10,21,23^. Therefore, approaches directly impacting monocyte/macrophage recruitment, macrophage activation, and/or macrophage functions and polarization are the most investigated^3,9^. Our data demonstrate that indirect targeting of macrophage phenotype through inhibition of MAIT cell activation may represent a promising alternative to promote fibrosis regression. We and others have shown that bidirectional crosstalk exists between MAIT cells and macrophages, since activated MAIT cells can switch macrophages toward a pro-inflammatory phenotype while pro-inflammatory macrophages can promote activation of MAIT cells, at least in part via TCR-MR1-dependent interactions^18,28,31^. Importantly, our findings further argue for a direct MAIT macrophage dialog and specifically demonstrate that activated MAIT cells increase the CCR2/CD206 macrophage ratio, an effect that is blunted by disrupting MAIT cell activation upon macrophage presentation of Ac-6-FP. We also demonstrate that inhibiting MAIT cell activation by Ac-6-FP affects both the frequency and the phenotype of Ly6C^hi^ vs Ly6C^lo^ macrophages. Indeed, there was a decrease in the frequency of Ly6C^hi^/CCR2+ MoMac and a concomitant increase in that of Ly6C^lo^ MoMac, when CCl_4_-exposed mice were injected Ac-6-FP after cessation of the injury. Interestingly, although Kupffer cells are the main population expressing MR1 in physiological conditions, their depletion in the fibrotic liver gave rise to Ly6C^hi^ and Ly6C^lo^ MoMac populations that upregulated MR1 at their cell surface. Finally, the use of clodronate liposomes in mice with established fibrosis depleted, at least in part, Ly6C^lo^ MoMac while it marginally affected Ly6C^hi^ MoMac. Interestingly, clodronate liposomes abolished the beneficial effect of Ac-6-FP on fibrosis regression, suggesting that restorative Ly6C^lo^ MoMac is required for Ac-6-FP beneficial effects. Taken together, these data also further reinforce that modulating the MAIT cell/macrophage dialog is required and sufficient for promoting fibrosis regression, independently of interactions with other liver cells such as hepatic stellate cells or hepatocytes.

RNA sequencing and functional studies also showed that Ac-6-FP profoundly modified the Ly6C^hi^ vs Ly6C^lo^ signature and caused deregulation of pathways related to cell cycle/apoptosis, metabolism, autophagy, and to a minor extent inflammation. In particular, and in keeping with the decrease in Ly6C^hi^ MoMac frequency, apoptosis was the most significant deregulated KEGG pathway affected by Ac-6-FP, with apoptotic genes mainly upregulated in Ly6C^hi^ and survival genes in Ly6C^lo^ counterparts. One of the proposed mechanisms underlying the shift from fibrogenic to resolutive macrophages is through a phenotypic switch^8^. Our data also suggest that enhancing apoptosis of Ly6C^hi^ MoMac, while sustaining Ly6C^lo^ could constitute an additional mechanism. In keeping, we have previously demonstrated that inducing apoptosis of pro-inflammatory monocyte/macrophages protects against chronic liver injury^32^.

Another anti-inflammatory and antifibrogenic pathway targeted by Ac-6-FP is autophagy, with the increase of a set of non-overlapping but essential autophagic genes both in Ly6C^hi^ and Ly6C^lo^ subtypes. The MR1-blocking ligand upregulated genes that contribute to different steps of the autophagic process, including autophagosome formation (*Pi3kc3, Atg14, Atg10*) and maturation (*Wipi1, Atg5/Atg16 and Atg16l2*). It should also be underlined that some of these autophagic genes, such as *Pik3c3, Atg5,* or *Atg16* also drive LC3-associated phagocytosis, a non-canonical form of autophagy with anti-inflammatory properties in macrophages^33^. These data were also confirmed in functional studies, which showed an increase in the frequency of LC3II-positive cells in both subtypes of macrophages. They are in line with the antifibrogenic properties of canonical and non-canonical autophagy in macrophages^19,33,34^.

Finally, Ac-6-FP also regulated genes related to lipid metabolism, mainly affecting lipid metabolic pathway in Ly6C^hi^ MoMac. Changes in lipid metabolism are increasingly recognized as a driving force for macrophage reprogramming, although data regarding scar-associated macrophages in the context of liver fibrosis are scarce^35^. RNA sequencing analysis revealed that Ac-6-FP mainly induced a large set of genes in Ly6C^hi^ MoMac related to glycerophospholipid metabolism, that converged to phosphatidic acid formation from glycerol-3 phosphate/LPA (*Gpd2, Gpcpd1, Gpat4, Agpact2, and 9*), diacylglycerol formation and degradation (*Dgka and z*) or lysophosphatidylcholine/phosphatidylcholine (*Lpcat1*). Interestingly, enhanced expression of genes affiliated with glycerophospholipid metabolic pathway may also provide a link between lipid metabolism and autophagy. Indeed, besides PI(3)P, recognized as a master regulator of autophagosome biogenesis and fusion, glycerophospholipids such as phosphatidic acid have also been reported to stimulate autophagosome biogenesis^24^, or reduce the inhibitory protein mTORC2 activity^36^. In addition, we also found that Ac-6-FP-exposed Ly6C^hi^ MoMac showed increased *Taz* gene expression, coding for tafazzin, which promotes maturation of cardiolipin, and allows initiation of mitophagy through interaction with LC3^37^.

In conclusion, our data demonstrate that inhibiting MAIT cells promotes scar resolution via reprogramming of macrophage profile. They open interesting perspectives for therapies promoting liver fibrosis regression, based on the use of MAIT cell inhibitory ligand-directed approaches.

## Methods

Our research complies with all relevant ethical regulations. Human study protocol was approved by the Institutional Review Board of Paris North Hospitals, Paris Cité University, AP-HP (N°CER-2021-88), and animal study protocol was approved by the Ministère de l’Enseignement Supérieur, de la Recherche et de l’Innovation (APAFIS #30284-2021030912208867).

### Human liver samples

Liver samples were obtained from patients from both genders with advanced fibrosis (F2-F4 Metavir score, *n* = 11) undergoing liver resection or liver transplantation at the digestive surgical department of Beaujon Hospital (Clichy, France). Patients’ clinical data are presented in Table 1. Patients’ age is between 33 and 73. All patients gave written consent to participate in the study. The study conformed to the ethical guidelines of the 1975 Declaration of Helsinki and was approved by the Institutional Review Board of Paris North Hospitals, Paris Cité University, AP-HP (N°CER-2021-88). Fresh liver specimens were examined by a pathologist and samples were collected at distance from the tumor (when present) and surgical margins.

### Human ex vivo PCLS

Fresh liver specimens were harvested and immediately kept on ice in a sterile University of Wisconsin solution (Belzer UW® Cold Storage Solution, Bridge to Life, BTLBUW-1000). Liver cores were generated from liver specimens using 8 mm diameter biopsy punches, embedded into 5% low-gelling-temperature agarose (Sigma-Aldrich) and mounted in a tissue slicer (automated vibrating blade microtome, Leica Biosystems VT1200 S) filled with Hanks Balanced Salt Solution supplemented with 25 mM of d-Glucose (Sigma-Aldrich), 100 μg/mL streptomycin, 1 μg/mL amphotericin B (Gibco^TM^). Precision-cut liver slices (PCLS, 8 mm diameter, 250 µm thickness) were generated using the following slicing parameters: speed 0.5 mm/s, thickness 250 µm, amplitude 3 mm. Human PCLS were transferred on 8 µm PET tissue culture inserts (ThinCert^TM^, Greiner bio-one) in six-well plates and pre-incubated in William’s E Medium (Gibco^TM^) supplemented with 100 IU/mL penicillin and 100 μg/mL streptomycin, 1 μg/mL amphotericin B (Gibco^TM^), and 25 mM d-Glucose (Sigma-Aldrich). After 1 h pre-incubation, fresh culture medium containing 10 µM Ac-6-FP or vehicle (0.01%DMSO) was added and PCLS were cultivated at 37 °C, 5% CO_2_, in normoxic conditions, under continuous orbital agitation (70 rpm). Culture medium was daily renewed. The viability of PCLS was assessed using PrestoBlue™ Cell Viability Reagent (Invitrogen, A13262) according to the manufacturer’s instructions (Fig. 1a, Table 1 Group 1) and was not affected by Ac-6-FP. After 48 h of culture, PCLS were washed in cold PBS and immediately snap frozen in liquid nitrogen and stored at −80 °C until processing for RNA extraction or fixed in 10% formalin for 24 h for immunohistochemistry experiments, as described below.

### Immunofluorescent staining of PCLS

Frozen sections (3µm-thick) generated from PCLS (*n* = 4, Table 1 Group 2) were fixed in 4% paraformaldehyde and further blocked in 1%BSA-PBS and 0.2% Triton X100 for 1 h at room temperature (RT). Slides were incubated with rabbit polyclonal anti-human CD69 antibody (1/50, #ab175391, Abcam) mouse anti-human Vα7.2 (3C10, 1/50, #351702, BioLegend) or mouse anti-human α-SMA (1A4, 1/1000, #A5228, Sigma-Aldrich) for 2 h at RT, followed by secondary antibody, either goat anti-rabbit Alexa488 (1/500, #A11034, Thermofisher Scientific), goat anti-mouse Alexa555 (1/200, #A28180, Thermofisher Scientific), or goat anti-mouse Alexa488 (1/1000, #A11001, Thermofisher Scientific) for 1 h at RT. PCLS were mounted by ProLong Gold Antifade Mounting medium with DAPI (Table 3). Representative images were acquired using a confocal microscope (Zeiss LSM 780), equipped with a ×63 oil immersion objective. For quantification, 20 to 28 fields were randomly selected, and the number of activated MAIT cells (CD69^+^Vα7.2^+^) and total MAIT cells (total Vα7.2^+^) was quantified. Results were expressed as % of CD69^+^Vα7.2^+^/total Vα7.2^+^ cells. No staining signal was observed when omitting the primary antibody.

**Table 3:** List of reagents for human experiments

	Supplier	Catalog #
Critical commercial assays		
Prestoblue™ cell viability reagent	Invitrogen	A13262
Immunohistochemistry antibody		
Mouse monoclonal anti-human α-SMA (1A4)	DAKO	M0851
Immunofluorescent antibodies		
Rabbit anti-human α-SMA (1A4)	Sigma-Aldrich	A5228
Mouse anti-human Vα7.2 (3C10)	BioLegend	351702
Polyclonal rabbit anti-human CD69	Abcam	ab175391
Goat anti-rabbit IgG secondary antibody Alexa488™	Thermofisher Scientific	A11034
Goat anti-mouse IgG secondary antibody Alexa555™	Thermofisher Scientific	A28180
Goat anti-mouse IgG secondary antibody Alexa488™	Thermofisher Scientific	A11001
Prolong® Diamond Antifade Mountant with DAPI	Thermofisher Scientific	P36962

### RNA extraction and quantitative polymerase chain reaction blot

RNA from PCLS (*n* = 7, Table 1 Group 1) was extracted in TRIzol™ reagent (Life Technologies) according to manufacturers’ instructions. cDNA synthesis was performed with QuantiTect Reverse Transcription Kit (Qiagen, 205313). Quantitative polymerase chain reaction (qPCR) was performed on Real-Time PCR system LightCycler® 96 Instrument (Roche) using TaqMan® probes for *ACTA2, CCL2, COL1A1*, *COL1A2*, *IL1B, TGFB1, TNFA, CCR2* and *PPIA* (#4331182, Applied Biosystems). Probes sequences are detailed in Table 2. PCR was performed with the following parameters: 1 cycle at 95 °C for 10 min followed by 45 cycles at 95 °C for 15 s, 60 °C for 30 s. Gene expression was normalized to *PPIA* housekeeping gene. Relative expression was calculated using the (2^−ΔΔCt^) method.

### Immunohistochemistry analysis of PCLS

Αlpha-smooth muscle actin (α-SMA) immunostaining was performed using formalin-fixed paraffin-embedded PCLS (*n* = 7 Table 1 Group 1) with an automated immunohistochemical stainer (Ventana Benchmark®) according to the manufacturer’s instructions. Briefly, 3 µm thickness tissue sections were dewaxed, rehydrated, and incubated with antibody targeting α-SMA (mouse monoclonal antibody, 1A4, 1/600, #M0851, DAKO, Table 3). Immunostained slides were digitized (Scanscope AT turbo®, Leica), and α-SMA staining was quantified using a dedicated algorithm (quantification of positive area, Indica labs) on the whole surface of each liver slice.

### Animals

B6-MAIT^CAST^ and MR1^−/−^ mice were obtained as previously reported^22^. Both strains have been backcrossed on a C57BL/6 J background for more than 10 generations. They were bred separately and housed in different cages in specific pathogen-free (SPF) animal facilities and fed *ad libitum*. Dark/light cycles were 12 h/12 h, with temperature of 21 °C and humidity of 50%. C57BL/6 J mice were purchased from Janvier Labs (C57BL/6 JRj). Cervical dislocation was used for mice euthanasia. Males were used for animal studies since it has been extensively reported that they are more susceptible to developing liver fibrosis than females (10.1152/ajpendo.00427.2019). Experiments were performed in accordance with protocols approved by the Ministère de l’Enseignement Supérieur, de la Recherche et de l’Innovation and the ethic committee APAFIS n°121 Paris Nord (APAFIS #30284-2021030912208867).

### Mouse models of liver fibrosis progression and regression

Liver fibrosis was induced in male C57BL/6J, B6-MAIT^CAST^, or MR1^−/−^ mice (10 to 12-week-old) by i.p injections of 0.6 ml/kg body weight (BW) carbon tetrachloride (CCl_4_, Sigma-Aldrich #87030, 1/10 dilution in mineral oil, Sigma-Aldrich #M-5310), twice a week for 4 weeks. Control animals received mineral oil (referred as to “oil” in the text) at the same frequency. To evaluate the impact of MAIT cell inhibition on fibrosis progression mice were administered CCl_4_ for 4 weeks with daily injection of Ac-6-FP (0.25 mg/kg body weight (b.w), #SMB01333, Sigma-Aldrich,) starting from 2.5 weeks (Fig. 2a).

To evaluate the impact of MAIT cell inhibition on fibrosis regression, two models were used. First, mice were injected intraperitoneally 2 h prior to the last CCl_4_ injection with MAIT cell inhibitors anti-MR1 (12.5 mg/kg b.w, 26.5, #361110, BioLegend), or Ac-6-FP, or their respective anti-IgG2a kappa isotype control (12.5 mg/kg b.w, #400281, BioLegend) or vehicle (1% DMSO in ×1 PBS) and daily until the sacrifice by cervical dislocation at day 1, day 2 or day 4 following the last CCl_4_ injection (Fig. 2d).

A second model of NASH-induced liver fibrosis consisted in 8 weeks choline-deficient l-amino acid defined High Fat Diet (CDAA-HFD) feeding (#A06071302i, Brogaarden, 60 kcal% fat, and 0.1% methionine, irradiated). Fibrosis regression was studied at 1 and 8 days after switching to normal diet, with daily injection of Ac-6-FP or its vehicle (Fig. 2c).

Timelines of injections shown in Fig. 2a, c, d were created with BioRender® software.

### Histological analysis

Sirius Red staining was performed on 4-μm-thick formalin-fixed paraffin-embedded tissue sections at the Pathology Department of Bichat Hospital, Paris. Sirius red-stained areas from ten fields (×10 magnification) of each mouse were quantified with the software ImageJ as previously described^18^.

### Immunohistochemistry analysis of mice liver

Immunohistochemical detection of α-SMA was performed as previously described^18^ (Table 4), on paraffin-embedded mice liver tissue sections (4 μm-thick) using the MOM immunodetection kit (Vector, #PK2002) and a mouse monoclonal anti-α-SMA antibody (1A4, 1/1000, #A2547, Sigma). α-SMA-positive area from ten fields (×10 magnification) from 5–8 mice/group was quantified with ImageJ software. No staining was observed when the primary antibody was omitted.

**Table 4:** List of reagents for mouse experiments

	Supplier	Catalog #
Critical commercial assays		
Liver Dissociation kit, mouse	Miltenyi	130-105-807
Guava Autophagy LC3 antibody-based detection kit	Luminex	FCCH100171
Dynabeads™ untouched™ mouse T cells kit	Thermofisher Scientific	11413D
Immunohistochemistry antibody		
Monoclonal anti-human/mouse α-SMA (1A4)	Sigma-Aldrich	A2547
In vivo antibodies		
Anti-MR1 (26.5)	BioLegend	361110
Anti-IgG2a kappa isotype control	BioLegend	400281
In vitro antibodies		
Ultra-LEAF™ purified anti-mouse IL-17A (TC11-18H10.1)	BioLegend	506945
Ultra-LEAF™ purified anti-mouse TNFα (MP6-XT22)	BioLegend	506332
Ultra-LEAF™ purified rat IgG1k isotype control (RTK2071)	BioLegend	400432
Ultra-LEAF™ purified anti-mouse CD3 (145-2C11)	BioLegend	100340
Ultra-LEAF™ purified anti-mouse CD28 (37.51)	BioLegend	102116
Immunofluorescent antibodies		
FITC rat anti-mouse CCR2 (SA203G11)	BioLegend	150608
Goat polyclonal anti-mouse CD206	Santa Cruz Biotechnology	sc-34577
Donkey anti-goat IgG secondary antibody Alexa555™	ThermoFisher scientific	A21432

### Macrophage depletion by clodronate liposomes

To deplete macrophages, C57BL/6 J mice with established fibrosis were given one i.v injection of liposome-encapsulated clodronate (Clodronate Liposomes, 0.1 ml/10 g, B#C23J0518, Liposoma). Liposome-encapsulated PBS was used as control (B#P24J0518, Liposoma). Ac-6-FP was then administered daily until the sacrifice at day 1, day 2, or day 4 following the last CCl_4_ injection. Timeline of injections shown in Fig. 3g were created with BioRender® software.

### Intrahepatic leukocyte isolation

Following perfusion of 1× PBS through the portal vein, livers were placed into DMEM Dulbecco’s Modified Eagle Medium (DMEM, ThermoFisher, Table 4) and digested using the Liver Dissociation kit (#130-105-807, Miltenyi), according to the manufacturer’s instructions. Digests were passed through 100 µm cell strainer and washed in 30 ml DMEM and centrifuged 300 × *g* for 10 min. Pellets were resuspended in a 33% Percoll (GE Healthcare) diluted with RPMI complete medium (RPMI 1640 from ThermoFisher containing 10% FBS) at room temperature and centrifuged 690 × *g* for 20 min, with minimum break and accelerator. The pellet, containing non-parenchymal cells, was washed with RPMI complete medium. Red blood cells were lysed using 5 ml RBC Lysis buffer (420301, BioLegend) for 3 min and washed with RPMI complete medium. The pelleted cells were resuspended in 2 ml FACS buffer (1× PBS supplemented with 2%FBS and 2 mM EDTA) and transferred to FACS tubes for staining.

### Ly6C^hi^ and Ly6C^lo^ macrophage sorting

C57BL/6 J mice with established fibrosis (0.6 ml/kg b.w CCl_4_ twice a week for 4 weeks), were i.p injected with Ac-6-FP (0.25 mg/kg b.w) or vehicle (1% DMSO in 1× PBS) 2 h prior to the last injection and daily during 2 days following the last CCl_4_ injection. Ly6G-CD11c-CD3-CD11b + F4/80 + Ly6C^hi^ or Ly6C^lo^ macrophages were sorted according to their Ly6C expression level using BD Biosciences FACSAria III sorter (Fig. S4a).

### Flow cytometric analysis

Cell suspensions prepared from non-parenchymal liver were stained at 4 °C in FACS buffer (1× PBS containing 2%FBS and 2 mM EDTA). Surface staining was performed with the following antibodies (Table 5) fluorochrome-conjugated anti-mouse: TCRβ (H57-597, 1/200, #109241), CD3 (17A2, 1/50, #100236), CD4 (RM4-5BL, 1/100, #100526), CD8 (53-6.7, 1/100, #100742), CD69 (H1.2F3, 1/100, #104509), F4/80 (BM8, 1/100, #123147), Ly6C (HK1.4, 1/200, #128041), CD19 (6D5, 1/200, #115553), CD11c (N418, 1/100, #117316), CCR2 (SA203G11, 1/100, #150613), NK1.1 (PK136, 1/50, #108727), and Tim-4 (RMT-54, 1/200, #130009) mAbs obtained from BioLegend, France; CD45 (30-F11, 1/200, #560510), CD11b (M1/70, 1/50, #560456), Ly6G (IA8, 1/200, #560602), and TCRγδ (GL3, 1/500, #563532) mAbs obtained from BD Biosciences. Dead cells were excluded from the analysis using the fixable viability dye eFluor 506 (1/200, #65-0866-14, eBiosciences).

**Table 5:** List of flow cytometry antibodies for mouse experiments

	Supplier	Catalog #
Flow cytometry antibodies		
BV605 anti-mouse TCRβ (H57-597)	BioLegend	109241
APC anti-mouse CD3 (17A2)	BioLegend	100236
APC-Cy7 anti-mouse CD4 (RAM4-5)	BioLegend	100526
BV650 anti-mouse CD8a (53-6.7)	BioLegend	100742
PE/Cy5 anti-mouse CD69 (H1.2F3)	BioLegend	104509
PE-CF594 anti-mouse TCRγδ (GL3)	BD Bioscience	563532
V450 anti-mouse CD11b (M1/70)	BD Bioscience	560456
BV785 anti-mouse Ly6C (HK1.1)	BioLegend	128041
BV711 anti-mouse F4/80 (BM8)	BioLegend	123147
PE-Cy7 anti-mouse Tim-4 (RMT-54)	BioLegend	130009
PE/Dazzle 594 anti-mouse CD19 (6D5)	BioLegend	115553
PerCP/CY5.5 anti-mouse Ly6G (IA8)	BD Bioscience	560602
PE-Cy5 anti-mouse CD11c (N418)	BioLegend	117316
AF700 anti-mouse CD45 (30-F11)	BD Bioscience	560510
PerCP/CY5.5 anti-mouse NK1.1 (PK136)	BioLegend	108727
BV650 anti-mouse CCR2 (SA203G11)	BioLegend	150613
PE anti-mouse MR1 (26.5)	BioLegend	361106
eFluor 506 Fixable Viability Dye	eBiosciences	65-0866-14
PE/Cy7 anti-mouse TNFα (MP6-XT22)	BioLegend	506324
PE anti-mouse IL-17A (TC11-19H10.1)	BioLegend	506904
APC-conjugated anti-human/mouse 5-OP-RU loaded MR1-tetramer	NIH	

Liver MAIT cells were identified in B6-MAIT^CAST^ by multicolor flow cytometry from CD45^+^CD19^−^CD11b^−^TCRβ^+^ as GFP+ CD4^−^CD8^−^ T cells. Similar findings were obtained when MAIT cells were gated with APC-conjugated mouse MR1 tetramers loaded with the active ligand 5-OP-RU (NIH, USA); APC-conjugated mouse MR1 tetramers loaded with the non-activating ligand Ac-6-FP were used as negative control “control tet” (Fig. S3). Data acquisition was performed using a BD Biosciences Fortessa X20 cytometer and analyzed using FlowJo software (Tree Star, 10.7.1 version).

### Intracellular staining

For detection of cytokine production in MAIT cells, intrahepatic leukocytes were isolated, and cells were stimulated for 4 h at 37 °C with 25 ng/ml PMA and 1 μg/ml ionomycin (Sigma-Aldrich), in the presence of 10 μg/ml Brefeldin A (BioLegend) in RPMI 1640 medium (Gibco) supplemented with 10% fetal bovine serum (Gibco). After surface staining, cells were fixed and permeabilized with Cytofix/Cytoperm kit (#554714, BD Biosciences), according to manufacturers’ instructions, washed and labeled for 30 min at 4 °C with anti-TNFα (MP6-TX22, 1/50, #506324), anti-IL17 (TC11-18H10, 1/50, #506904), from BioLegend. Detection of LC3II in Ly6C^hi^ and Ly6C^lo^ macrophages was performed by flow cytometry with the Guava Autophagy LC3 antibody-based detection kit (FCCH100171, Luminex, Table 4), according to the manufacturer’s instructions.

### RNA isolation and RNA-Seq analysis

Following Ly6C^hi^ and Ly6C^lo^ macrophage sorting, cells were directly frozen at −80 °C and RNA extracted extemporaneously using RNeasy Micro kit (74004, QIAgen). RNAs were qualified and quantified with AGILENT Tapestation 2200 on High Sensitivity RNA chip. RNA library preparation was realized following manufacturer’s recommendations (NEBNext Single Cell/Low Input RNA Library Prep from New england Biolabs). cDNA and final libraries were controlled with AGILENT Tapestation 2200 on High Sensitivity DNA chip. Final equimolar pooled library preparations were sequenced on Nextseq 500 ILLUMINA with HighOutPut cartridge (2x400Millions of 75 bases reads). Corresponding to 2x20Millions of reads per sample after demultiplexing.

RNA-Seq data analysis was performed by GenoSplice technology (www.genosplice.com). Sequencing, data quality, reads repartition (e.g., for potential ribosomal contamination), and inner distance estimation was performed using FastQC, Picard-Tools, Samtools, and RSeQC. Reads were mapped using STARv2.4.0^38^ on the mm10 Mouse genome assembly. Gene expression regulation study was performed as already described^39–42^. Briefly, for each gene present in the Mouse FAST DB v2018_1 annotations, reads aligning on constitutive regions (that are not prone to alternative splicing) were counted. Based on these read counts, normalization was performed using DESeq2^43^. Genes were considered as expressed if their FPKM value was greater than 95% of the background FPKM value based on intergenic regions defined from the Mouse FAST DB v2018_1 annotations. Only genes expressed in at least one of the two compared experimental conditions were further analyzed.

DESeq2 normalized counts were log2 transformed and each Ac-6-FP treated sample was normalized according to the mean between the corresponding VEH log2 normalized counts (i.e., Ly6C^hi^ Ac-6-FP and Ly6C^lo^ Ac-6-FP samples were normalized to respective mean vehicle samples). A paired *t* test was performed between Ly6C^hi^ Ac-6-FP/mean vehicle ratios and Ly6C^lo^ Ac-6-FP/mean vehicle ratios. Results were considered statistically significant for uncorrected *p* values ≤0.05 and fold-changes ≥1.5. Enrichment analyses were performed using WebGestaltR^44^ on KEGG pathways. The sequence datasets have been deposited in the Gene Expression Omnibus with the accession number GSE183906 (Supplementary data 1) [https://www.ncbi.nlm.nih.gov/geo/query/acc.cgi?acc=GSE183906].

### Co-cultures of MAIT cell and macrophages and immunostaining

MAIT cells were isolated from the spleen of B6-MAIT^CAST^ mice using Dynabeads™ untouched™ mouse T cells kit, according to the manufacturer’s instructions. MAIT cells were then sorted as CD3^+^CD4^−^CD8^−^GFP^+^ cells using BD FACSMelody™ cell sorter, following staining with flow cytometry antibodies as described above (Fig. S5). MAIT cells were pre-activated overnight with 10 μg/ml anti-CD3 (145-2C11, #100340) and 2.5 μg/ml anti-CD28 (37.51, #102116) from BioLegend. BMDM were isolated from C57BL/6 J mice femur and tibia bone marrow, differentiated for 7 days in RPMI containing 10%FCS and 1% penicillin/streptomycin, in the presence of L929 media containing M-CSF. BMDM (3 × 10^4^ cells/well) were seeded in μ-slide 18 well ibiTreat (iBidi, 81816) and co-cultured with MAIT (3 × 10^4^ cells/well) for 48 h in the presence of 10 μM of Ac-6-FP or its vehicle, with a 1:1 MAIT:Macrophage ratio. Cytokine-neutralizing experiments were performed by adding 10 μg/ml of mouse anti-IL-17A (TC11-18H10.1, #506945, BioLegend) and/or anti-TNFα (MP6-XT22, #506332, BioLegend) or isotype (RTK2071, #400432, BioLegend) to the culture medium. BMDM were then fixed by 4% paraformaldehyde and blocked in 1%BSA-PBS and 0.2% Triton X100 for 1 h at RT. Cells were incubated with either FITC rat anti-mouse CCR2 (SA203G11, 1/50, #150608, BioLegend) or goat polyclonal anti-mouse CD206 (1/50, #sc-34577, Santa Cruz Biotechnology) for 2 h at RT, followed by donkey anti-goat Alexa555 (1/500, #A21432, Thermofisher Scientific) for 30 min (Table 4). Cells were mounted by mounting medium with DAPI (50011, ibidi). Representative images were acquired using confocal microscope (Zeiss LSM 780). For quantification, 10–12 fields were randomly selected, and mean intensity of CCR2 and CD206 per cell were measured by Image J. No staining signal was observed when omitting the primary antibody. Cells were isolated from female and male mice.

### Statistical analysis

The results are expressed as mean ± standard error of the mean (SEM) or median (interquartile range), as indicated. Mann–Whitney test was used to calculate significant levels between the two groups. For comparison of means from multiple groups against one control group, one-way ANOVA followed by Bonferroni’s multiple comparisons post-test analysis was performed. All *p* values are two-sided, and *p*-values less than 0.05 were considered to be statistically significant. Each variable achieving a *p* value < 0.05 was then introduced into a bivariate model. Wilcoxon matched-pairs signed rank test was used to assay the statistical significance of Ac-6-FP treatment on human PCLS compared to vehicle. All the analyses were performed using GraphPad Prism 8.4.3 software (GraphPad Software, Inc., La Jolla, CA). Sample sizes were adequate to detect large effects between groups, as determined by the reproducibility and variability of each particular experiment and limited by the availability of animal samples.

### Reporting summary

Further information on research design is available in the Nature Portfolio Reporting Summary linked to this article.

## Supplementary information

Supplementary Information

Description of Additional Supplementary Files

Supplementary Data 1

Reporting Summary

## Data Availability

The sequence datasets have been deposited in the Gene Expression Omnibus under accession code GSE183906. Other data that support the findings of this study are available from the corresponding author upon reasonable request. Source data are provided with this paper.

## Source data

Source Data
