# Supplementary material for: MAIT cell inhibition promotes liver fibrosis regression via macrophage phenotype reprogramming
Source: Nat Commun. 2023 Apr 1;14:1830. doi: 10.1038/s41467-023-37453-5 (PMC10067815; doi:10.1038/s41467-023-37453-5)
Supplement: Supplementary file 1 — Supplementary Information [file 41467_2023_37453_MOESM1_ESM.pdf]

MAIT cell inhibition promotes liver fibrosis regression via macrophage phenotype reprogramming

Morgane Mabire[1], Pushpa Hegde[1], Adel Hammoutene[1$], Jinghong Wan[1$], Charles Caër [1$], Rola Al Sayegh[1], Mathilde Cadoux[1], Manon Allaire[1], Emmanuel Weiss[1,2], Tristan Thibault-Sogorb[1,2], Olivier Lantz[3], Michèle Goodhardt[4], Valérie Paradis[1,5], Pierre de la Grange[6], Hélène Gilgenkrantz[1], Sophie Lotersztajn[1*]

Supplementary Files

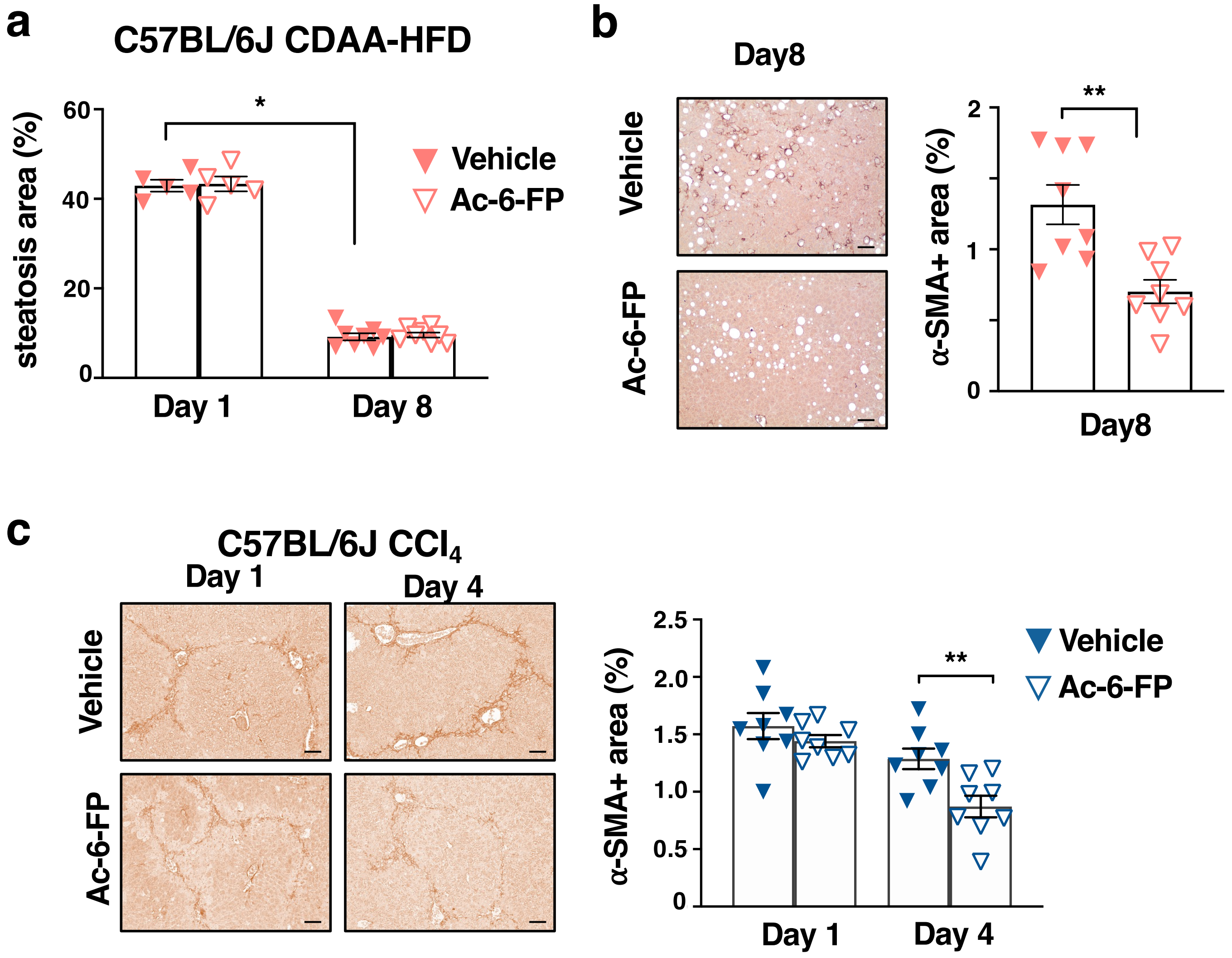
a
C57BL/6J CDAA-HFD
steatosis area (%)
60
40
20
0
*
Vehicle
Ac-6-FP
Day 1
Day 8
b
Day8
Vehicle
Ac-6-FP
α-SMA+ area (%)
2
1
0
**
Day8
c
C57BL/6J CCl4
Day 1
Day 4
Vehicle
Ac-6-FP
α-SMA+ area (%)
2.5
2.0
1.5
1.0
0.5
0
**
Vehicle
Ac-6-FP
Day 1
Day 4

Figure S1

Figure S1. Blocking MAIT cell activation accelerates liver fibrosis regression. CDAA-HFD fed C57BL/6J mice and CCl_4-injected C57BL/6J mice were processed as in **Fig 2c** and **d**. Representative images and quantification of (**a**) steatosis areas and (*p=0.01) (**b**) α-SMA positive areas in liver tissue sections from CDAA-HFD fed mice (n=5 mice/group at day 1; n=8 mice/group at day 8; **p=0.005). (**c**) Representative images and quantification of Sirius red areas in liver tissue sections from CCl_4-injected mice (n=8 mice/group; **p=0.005). Data are mean ± S.D. Statistical analysis were performed by (**a)** Kruskal-Wallis followed by Dunn's multiple comparisons post-test, **(b, c**) two-tailed Mann-Whitney test.

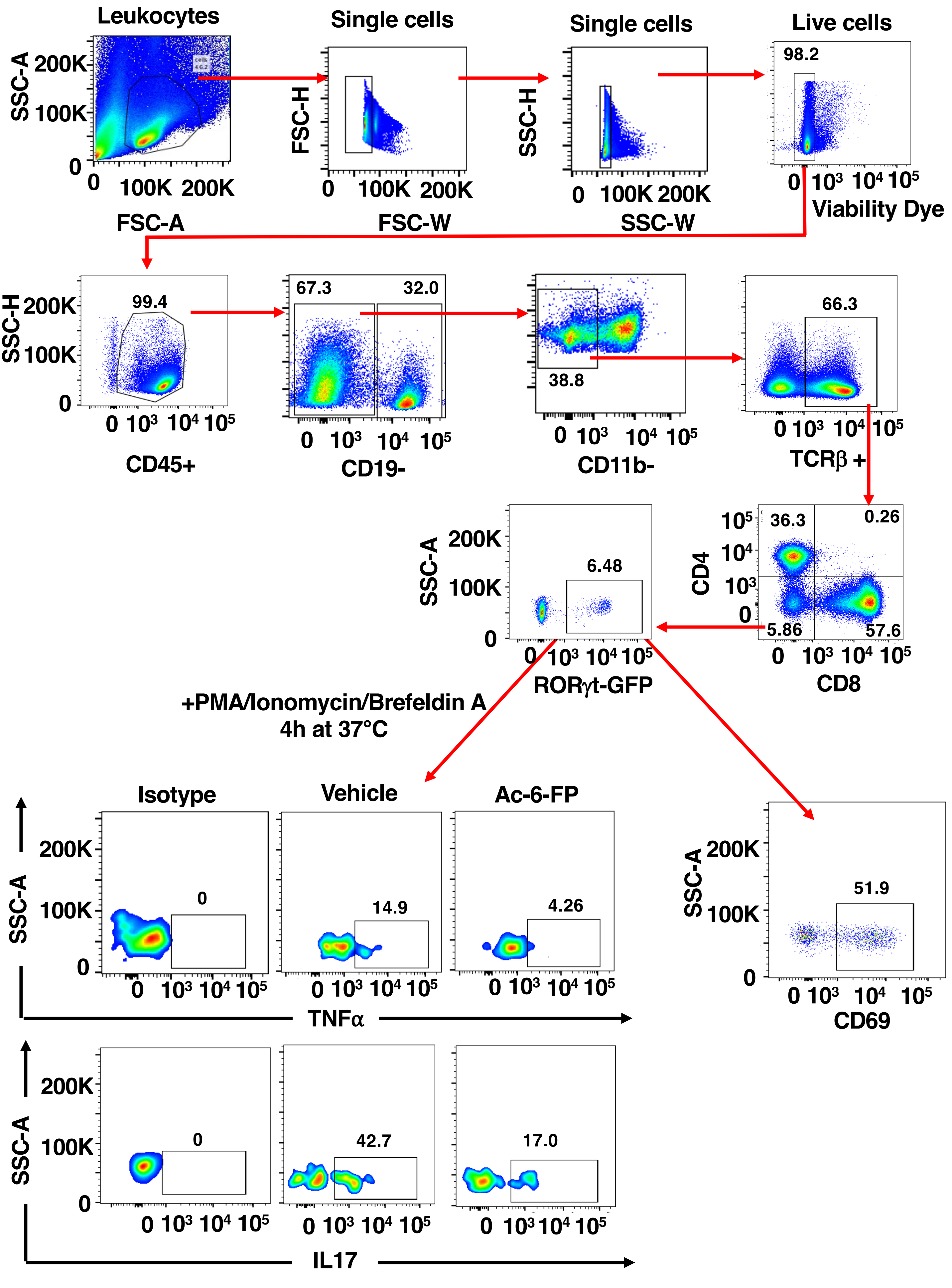
Leukocytes
Single cells
Single cells
Live cells
FSC-A
FSC-W
SSC-W
Viability Dye
98.2
99.4
67.3
32.0
38.8
66.3
CD45+
CD19-
CD11b-
TCRβ +
6.48
36.3
0.26
5.86
57.6
RORγt-GFP
CD8
CD4
+PMA/Ionomycin/Brefeldin A
4h at 37°C
Isotype
Vehicle
Ac-6-FP
0
14.9
4.26
TNFα
51.9
CD69
0
42.7
17.0
IL17
SSC-A
SSC-H
FSC-H

Figure S2

Figure S2. Gating strategy of intrahepatic mouse MAIT cells. Leukocytes were isolated from CCl_4-injected B6-MAIT^{CAST} mice exposed to Ac-6-FP or vehicle. Representative gating strategy of liver MAIT cells, surface expression of CD69 and TNFα and IL17 cytokine profiles. TNFα+ and IL17+ cells were analyzed following leukocyte stimulation with PMA/Ionomycin for 4h.

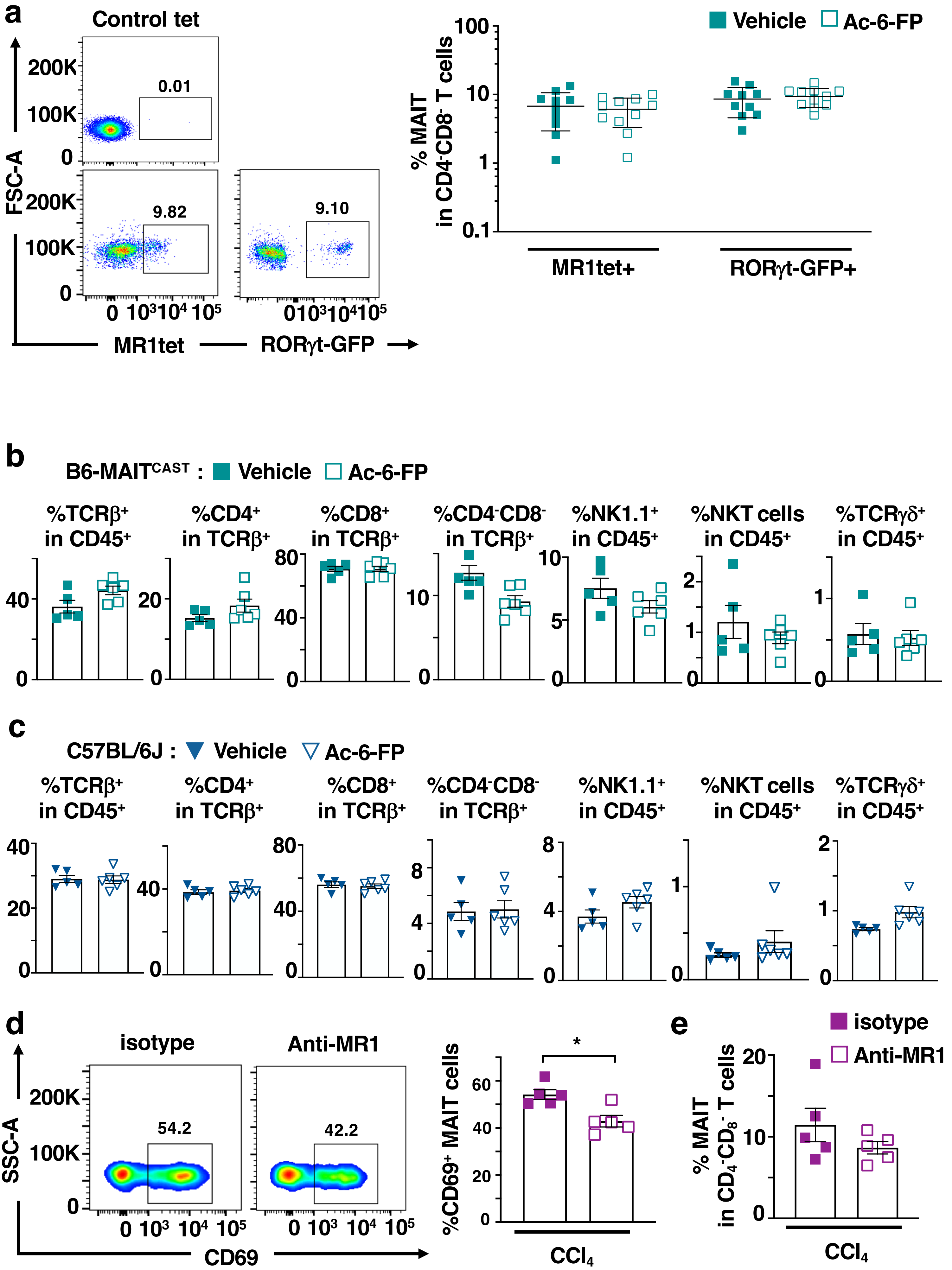
a
Control tet
0.01
9.82
9.10
FSC-A
MR1tet
RORγt-GFP
Vehicle
Ac-6-FP
% MAIT in CD4-CD8- T cells
MR1tet+
RORγt-GFP+
b
B6-MAITCAST : Vehicle Ac-6-FP
%TCRβ+ in CD45+
%CD4+ in TCRβ+
%CD8+ in TCRβ+
%CD4-CD8- in TCRβ+
%NK1.1+ in CD45+
%NKT cells in CD45+
%TCRγδ+ in CD45+
c
C57BL/6J : Vehicle Ac-6-FP
%TCRβ+ in CD45+
%CD4+ in TCRβ+
%CD8+ in TCRβ+
%CD4-CD8- in TCRβ+
%NK1.1+ in CD45+
%NKT cells in CD45+
%TCRγδ+ in CD45+
d
isotype
Anti-MR1
54.2
42.2
SSC-A
CD69
%CD69+ MAIT cells
*
CCl4
e
isotype
Anti-MR1
% MAIT in CD4-CD8- T cells
CCl4

Figure S3

Figure S3. MR1 blocking agents specifically target MAIT cells. CCl_4-injected B6-MAIT^{CAST} or C57BL/6J mice were injected once with Ac-6-FP, anti-MR1, vehicle or isotype after cessation of CCl_4 administration along the protocol described in **Fig 2d**. (**a**) Representative dot plots of liver MAIT cells gated either by MR1tet or by RORγtGFP (left) and respective quantification (right). Ac-6-FP loaded tetramers were used as negative control ("control tet") as compared to 5-OP-RU loaded tetramers ("MR1tet"). MAIT cell frequencies are expressed among CD4-CD8- T cells. Data were pooled from 2 different experiments (n= 11 for CCl_4-injected B6-MAIT^{CAST} mice exposed to Ac-6-FP and n=10 for vehicle). (**b, c**) Graphs showing the frequency of intrahepatic TCRβ+, NK1.1+, TCRγδ+ and NKT cells among CD45+, and CD4+, CD8+ and CD4-CD8- among TCRβ+ cells in CCl_4-injected (**b**) B6-MAIT^{CAST} and (**c**) C57BL/6J mice exposed to Ac-6-FP (n=6) or vehicle (n=5). (**d**) Representative dot plots and quantification of CD69+ MAIT cells in B6-MAIT^{CAST} mice exposed to MR1 antibody or isotype (n=5 mice/group). (**e**) Graph showing MAIT cell frequency among CD4-CD8- T cells in B6-MAIT^{CAST} mice exposed to MR1 antibody or isotype (n=5 mice/group; *p=0.03). Data are mean ± S.D. Statistical analysis were performed by two-tailed Mann-Whitney test.

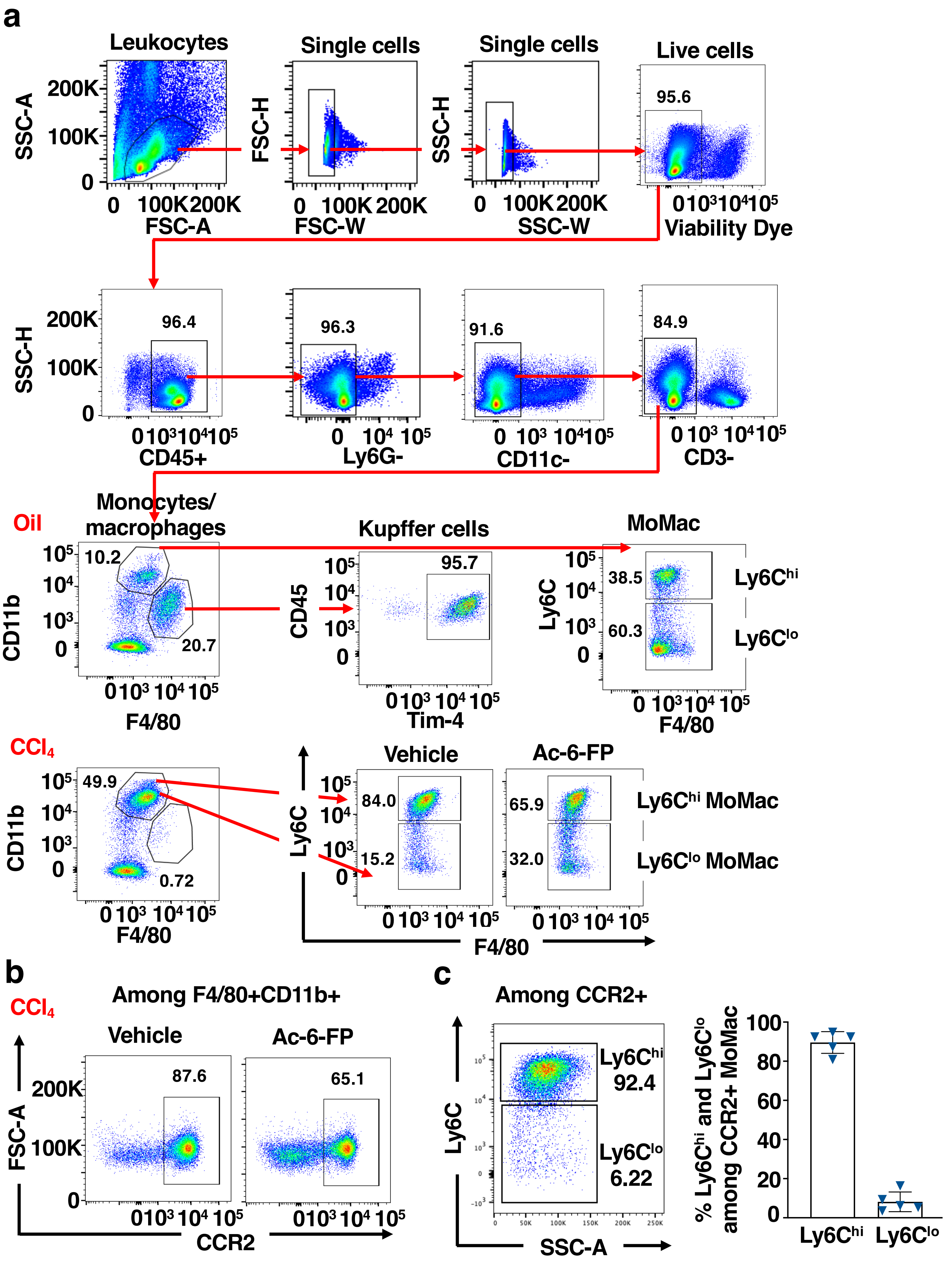
a
Leukocytes
Single cells
Single cells
Live cells
SSC-A
FSC-H
SSC-H
FSC-A
FSC-W
SSC-W
Viability Dye
95.6
CD45+
Ly6G-
CD11c-
CD3-
96.4
96.3
91.6
84.9
Oil
Monocytes/ macrophages
Kupffer cells
MoMac
CD11b
F4/80
CD45
Tim-4
Ly6C
10.2
20.7
95.7
38.5
60.3
Ly6Chi
Ly6Clo
CCl4
49.9
0.72
Vehicle
Ac-6-FP
84.0
15.2
65.9
32.0
Ly6Chi MoMac
Ly6Clo MoMac
b
CCl4
Among F4/80+CD11b+
Vehicle
Ac-6-FP
87.6
65.1
FSC-A
CCR2
c
Among CCR2+
Ly6Chi 92.4
Ly6Clo 6.22
SSC-A
% Ly6Chi and Ly6Clo among CCR2+ MoMac
Ly6Chi
Ly6Clo

Figure S4

Figure S4. Intrahepatic monocyte/macrophages gating strategy. CCl_4-exposed B6-MAIT^{CAST} or C57BL/6J mice were injected once with Ac-6-FP or vehicle after cessation of CCl_4 administration along the protocol described in **Fig 2d**. (**a**) Representative gating strategy of CD11b+F4/80+ MoMac and F4/80+Tim-4+ Kupffer cells from mice intrahepatic leucocytes. (**b**) Representative dot plots of CCR2+ cells among CD11b+F4/80+ MoMac in Ac-6-FP- or vehicle-injected mice. (**c**) Representative dot plot and quantification of Ly6C^{hi} and Ly6C^{lo} expression from CCR2+ MoMac; n=5 mice/group. Data are mean ± S.E.M.

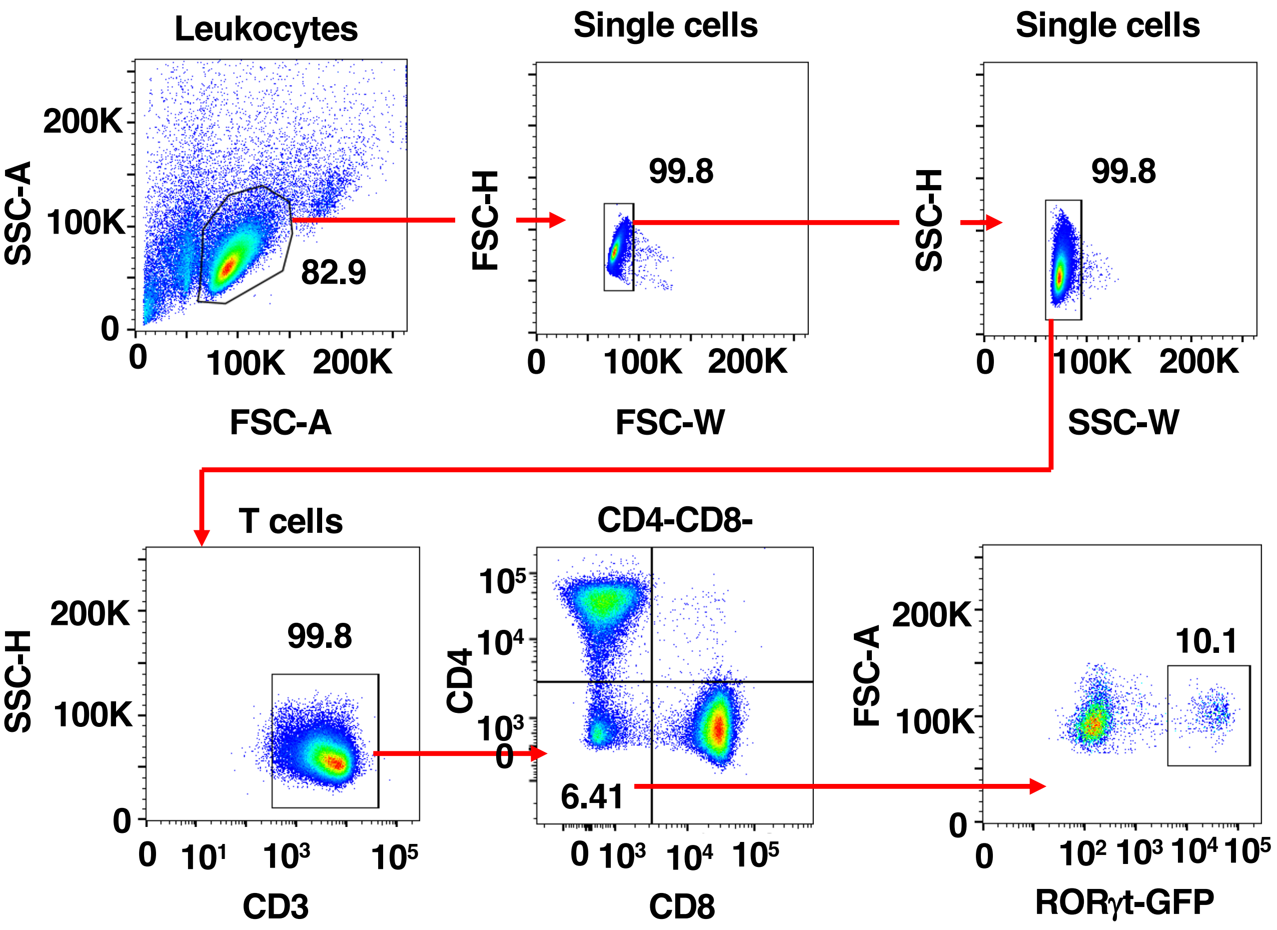
Leukocytes
Single cells
Single cells
SSC-A
200K
100K
0
82.9
FSC-A
FSC-H
99.8
FSC-W
SSC-H
99.8
SSC-W
100K
200K
T cells
CD4-CD8-
SSC-H
99.8
CD3
CD4
6.41
CD8
FSC-A
10.1
RORγt-GFP

Figure S5

Figure S5. Gating strategy of spleen mouse MAIT cells. Immune cells were isolated from spleen of CCl_4-injected B6-MAIT^{CAST} mice. Cell suspension was T cell enriched with Dynabeads™ untouched™ mouse T cells kit and MAIT cells were sorted based on this gating strategy as CD3+CD4-CD8-GFP+ cells.

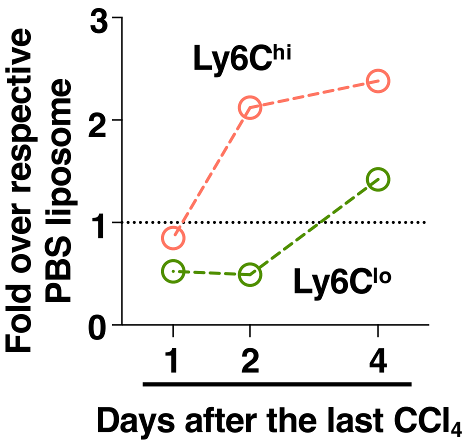
Fold over respective PBS liposome
3
2
1
0
Ly6Chi
Ly6Clo
1
2
4
Days after the last CCl4

Figure S6

Figure S6. Ac-6-FP does not impact the Ly6C^{hi} and Ly6C^{lo} depletion by clodronate liposomes. Clodronate vs PBS liposomes fold change of Ly6C^{hi} and Ly6C^{lo} MoMac frequencies in Ac-6-FP-injected mice ats day 1, 2 and 4 after CCl4 cessation along the protocol described in **Fig 3g**. Each point represents the mean of 5 mice.

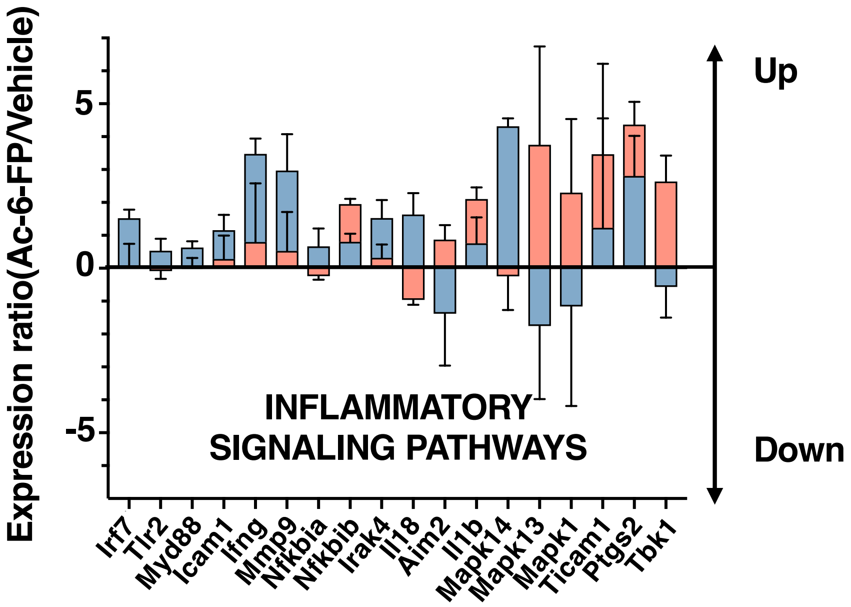
Expression ratio(Ac-6-FP/Vehicle)
5
0
-5
INFLAMMATORY
SIGNALING PATHWAYS
Up
Down
Irf7
Tlr2
Myd88
Icam1
Ifng
Mmp9
Nfkbia
Nfkbib
Irak4
Il18
Aim2
Il1b
Mapk14
Mapk13
Mapk1
Ticam1
Ptgs2
Tbk1

Figure S7

Figure S7. Blocking MAIT cell activation does not impact on the Ly6C^{hi} vs Ly6C^{lo} inflammatory signature. RNAseq analysis of Ac-6-FP/mean vehicle ratios in Ly6C^{hi} (red) and Ly6C^{lo} (blue) for selected genes from inflammatory signaling KEGG pathways that originated from Malaria (mmu05144), Hepatitis B (mmu05161), Leishmaniasis (mmu05140), NOD-like receptor signaling pathway (mmu04621), T cell receptor signaling pathway (mmu04660) and Measles (mmu05162). Data are presented as mean values ± S.E.M.
