# Supplementary material for: MAIT cell inhibition promotes liver fibrosis regression via macrophage phenotype reprogramming
Source: Nat Commun. 2023 Apr 1;14:1830. doi: 10.1038/s41467-023-37453-5 (PMC10067815; doi:10.1038/s41467-023-37453-5)
Supplement: Supplementary file 2 — Description of Additional Supplementary Files [file 41467_2023_37453_MOESM2_ESM.pdf]

Description of Additional Supplementary Files

File Name: Supplementary Data 1
Description: List of the 2,687 genes from the gene expression ratio analysis
