# Supplementary material for: MAIT cell inhibition promotes liver fibrosis regression via macrophage phenotype reprogramming
Source: Nat Commun. 2023 Apr 1;14:1830. doi: 10.1038/s41467-023-37453-5 (PMC10067815; doi:10.1038/s41467-023-37453-5)
Supplement: Supplementary file 4 — Reporting Summary [file 41467_2023_37453_MOESM4_ESM.pdf]

nature portfolio

Corresponding author(s): Sophie LOTERSZTAJN

Last updated by author(s): Feb 20, 2023

Reporting Summary

Nature Portfolio wishes to improve the reproducibility of the work that we publish. This form provides structure for consistency and transparency in reporting. For further information on Nature Portfolio policies, see our Editorial Policies and the Editorial Policy Checklist.

Statistics

For all statistical analyses, confirm that the following items are present in the figure legend, table legend, main text, or Methods section.

n/a	Confirmed	
☐	☒	The exact sample size (*n*) for each experimental group/condition, given as a discrete number and unit of measurement
☐	☒	A statement on whether measurements were taken from distinct samples or whether the same sample was measured repeatedly
☐	☒	The statistical test(s) used AND whether they are one- or two-sided<br>*Only common tests should be described solely by name; describe more complex techniques in the Methods section.*
☒	☐	A description of all covariates tested
☐	☒	A description of any assumptions or corrections, such as tests of normality and adjustment for multiple comparisons
☐	☒	A full description of the statistical parameters including central tendency (e.g. means) or other basic estimates (e.g. regression coefficient) AND variation (e.g. standard deviation) or associated estimates of uncertainty (e.g. confidence intervals)
☐	☒	For null hypothesis testing, the test statistic (e.g. *F*, *t*, *r*) with confidence intervals, effect sizes, degrees of freedom and *P* value noted<br>*Give P values as exact values whenever suitable.*
☒	☐	For Bayesian analysis, information on the choice of priors and Markov chain Monte Carlo settings
☒	☐	For hierarchical and complex designs, identification of the appropriate level for tests and full reporting of outcomes
☒	☐	Estimates of effect sizes (e.g. Cohen's *d*, Pearson's *r*), indicating how they were calculated

Our web collection on statistics for biologists contains articles on many of the points above.

Software and code

Policy information about availability of computer code

Data collection	GraphPad Prism software version 8.4.3, FlowJo analysis software V10.7 (Tree Star), Image J software version 1.53 and BioRender.com were used
Data analysis	Statistical analyses were performed using GraphPad Prism software version 8.4.3, ID custom : A57AF710E62<br>Flow cytometric analyses were performed with FlowJo analysis software V10.7 (Tree Star) , session ID : 107446045293758

For manuscripts utilizing custom algorithms or software that are central to the research but not yet described in published literature, software must be made available to editors and reviewers. We strongly encourage code deposition in a community repository (e.g. GitHub). See the Nature Portfolio guidelines for submitting code & software for further information.

Data

Policy information about availability of data

All manuscripts must include a data availability statement. This statement should provide the following information, where applicable:

- Accession codes, unique identifiers, or web links for publicly available datasets
- A description of any restrictions on data availability
- For clinical datasets or third party data, please ensure that the statement adheres to our policy

The sequence datasets have been deposited in the Gene Expression Omnibus with the accession number GSE183906, accessible with this link : https://

www.ncbi.nlm.nih.gov/geo/query/acc.cgi?acc=GSE183906. Others data that support the findings of this study are available from the corresponding author upon reasonable request. Source data are provided with this paper.

Human research participants

Policy information about studies involving human research participants and Sex and Gender in Research.

Reporting on sex and gender	Samples were collected from men and women depending on the availability of the liver explants, independently of the sex. 8 men and 3 women were included in this study
Population characteristics	All the patient's characteristics were described in Table 1. Age of the patients is between 33 and 73.
Recruitment	Patients were recuited according to the etiology and the severity of liver disease. Sex and age were rendom. However, patients undergoing surgery for these liver diseases are mainly aged men.
Ethics oversight	The protocol was approved by the Institutional Review Board of Paris North Hospitals, Paris Cité University, AP-HP (N° CER-2021-88)

Note that full information on the approval of the study protocol must also be provided in the manuscript.

Field-specific reporting

Please select the one below that is the best fit for your research. If you are not sure, read the appropriate sections before making your selection.

☒ Life sciences ☐ Behavioural & social sciences ☐ Ecological, evolutionary & environmental sciences

For a reference copy of the document with all sections, see nature.com/documents/nr-reporting-summary-flat.pdf

Life sciences study design

All studies must disclose on these points even when the disclosure is negative.

Sample size	For human studies size sample was based on our previous study on MAIT cells in liver fibrosis (Hegde et al, Nat Commun, 2018)<br>For mouse studies, the sample size was based on our previous study on MAIT cells in liver fibrosis (Hegde et al, Nat Commun, 2018 ; Habib et al, Gut, 2019 ; Wan et al, Science Trans Med, 2020)
Data exclusions	No
Replication	Data were reproduced in PCLS from n=11 patients. For mouse studies, at least two independent experiments were performed with at least n=5 mice/group. All the replicate experiments gave the same expected result.
Randomization	C57BL6/J, B6-MAIT CAST and MR1-/- mice were rendomized in the cages at weaning (30 days of age). Only male mice were used, at 10 to 12-week old, randomly allocated into experimental groups
Blinding	All experiments in human and mice were performed blinded

Reporting for specific materials, systems and methods

We require information from authors about some types of materials, experimental systems and methods used in many studies. Here, indicate whether each material, system or method listed is relevant to your study. If you are not sure if a list item applies to your research, read the appropriate section before selecting a response.

Materials & experimental systems

n/a	Involved in the study	
☐	☒	Antibodies
☒	☐	Eukaryotic cell lines
☒	☐	Palaeontology and archaeology
☐	☒	Animals and other organisms
☒	☐	Clinical data
☒	☐	Dual use research of concern

Methods

n/a	Involved in the study	
☒	☐	ChIP-seq
☐	☒	Flow cytometry
☒	☐	MRI-based neuroimaging

Antibodies

Antibodies used

All antibodies were commercial and already used by us and many investigators for several years. They are described in Table 3, 4 and 5, and in the methods. We didn't use any undescribed antibody.
Surface
staining was performed with the following antibodies :

Rabbit anti-human a-SMA (1A4) Sigma-Aldrich A5228
Mouse anti-human Va7.2 (3C10) BioLegend 351702
Polyclonal rabbit anti-human CD69 Abcam ab175391
Goat anti-rabbit IgG secondary antibody Alexa488™ Thermofisher Scientific A11034
Goat anti-mouse IgG secondary antibody Alexa555™ Thermofisher Scientific A28180
Goat anti-mouse IgG secondary antibody Alexa488™ Thermofisher Scientific A11001
Mouse monoclonal anti-human a-SMA (1A4) DAKO M0851

Monoclonal anti-human/mouse a-SMA (1A4) Sigma-Aldrich A2547

Anti-MR1 (26.5) BioLegend 361110
Anti-IgG2a kappa isotype control BioLegend 400281

Ultra-LEAF™ purified anti-mouse IL-17A (TC11-18H10.1) BioLegend
506945
Ultra-LEAF™ purified anti-mouse TNFα (MP6-XT22) BioLegend 506332
Ultra-LEAF™ purified rat IgG1k isotype control (RTK2071) BioLegend 400432
Ultra-LEAF™ purified anti-mouse CD3 (145-2C11) BioLegend 100340
Ultra-LEAF™ purified anti-mouse CD28 (37.51) BioLegend 102116

FITC rat anti-mouse CCR2 (SA203G11) BioLegend 150608
Goat polyclonal anti-mouse CD206 Santa Cruz Biotechnology sc-34577
Donkey anti-goat IgG secondary antibody Alexa555™ ThermoFisher scientific A21432
BV605 anti-mouse TCR (H57-597) BioLegend 109241
APC anti-mouse CD3 (17A2) BioLegend 100236
APC-Cy7 anti-mouse CD4 (RAM4-5) BioLegend 100526
BV650 anti-mouse CD8a (53-6.7) BioLegend 100742
PE/Cy5 anti-mouse CD69 (H1.2F3) BioLegend 104509
PE-CF594 anti-mouse TCR (GL3) BD Bioscience 563532
V450 anti-mouse CD11b (M1/70) BD Bioscience 560456
BV785 anti-mouse Ly6C (HK1.1) BioLegend 128041
BV711 anti-mouse F4/80 (BM8) BioLegend 123147
PE-Cy7 anti-mouse Tim-4 (RMT-54) BioLegend 130009
PE/Dazzle 594 anti-mouse CD19 (6D5) BioLegend 115553
PerCP/CY5.5 anti-mouse Ly6G (IA8) BD Bioscience 560602
PE-Cy5 anti-mouse CD11c (N418) BioLegend 117316
AF700 anti-mouse CD45 (30-F11) BD Bioscience 560510
PerCP/CY5.5 anti-mouse NK1.1 (PK136) BioLegend 108727
BV650 anti-mouse CCR2 (SA203G11) BioLegend 150613
PE anti-mouse MR1 (26.5) BioLegend 361106
eFluor 506 Fixable Viability Dye eBiosciences 65-0866-14
PE/Cy7 anti-mouse TNF (MP6-XT22) BioLegend 506324
PE anti-mouse IL-17A (TC11-19H10.1) BioLegend 506904
APC-conjugated anti-human/mouse 5-OP-RU loaded MR1-tetramer. MR1 tetramers were generated by A.Corbett and J.McCluskey and the NIH facility

Validation

Rabbit anti-human a-SMA (1A4) Sigma-Aldrich A5228. Ref to the publication: van Royen, N., et al., FASEB, 16, 432-434 (2002)
Mouse anti-human Va7.2 (3C10) BioLegend 351702. Ref to the publication: Radtke AJ, et al. 2022. Nat Protoc. 17:378-401
Polyclonal rabbit anti-human CD69 Abcam ab175391. Ref to the publication: PMID : 33240271
Goat anti-rabbit IgG secondary antibody Alexa488™ Thermofisher Scientific A11034. Ref to the publication: DOI: 10.26508/lsa.202201616
Goat anti-mouse IgG secondary antibody Alexa555™ Thermofisher Scientific A28180. Ref to the publication: DOI: 10.1186/s13578-022-00923-2
Goat anti-mouse IgG secondary antibody Alexa488™ Thermofisher Scientific A11001. Ref to the publication: DOI: 10.1083/jcb.202204102 10.4103/1673-5374.353493
Mouse monoclonal anti-human a-SMA (1A4) DAKO M0851. Ref to the publication: Brennan PA, et al. J Oral Pathol Med 2000,29:279-83.

Monoclonal anti-human/mouse a-SMA (1A4) Sigma-Aldrich A2547. Ref to the publication: Skalli, O., et al., J. Cell Biol., 103, 2787 (1986).

Anti-MR1 (26.5) BioLegend 361110. Ref to the publication: Kjer-Nielsen L, et al. 2012. Nature 491:717. (Block, ELISA)
Anti-IgG2a kappa isotype control BioLegend 400281. Ref to the publication: Montes de Oca M, et al. 2016. Cell Rep. 17:399-412

Ultra-LEAF™ purified anti-mouse IL-17A (TC11-18H10.1) BioLegend 506945. Ref to the publication: Cui Y, et al. 2009. Invest. Ophth.

Vis. Sci. 50:5811
Ultra-LEAF™ purified anti-mouse TNFα (MP6-XT22) BioLegend 506332. Ref to the publication: McKnight Q, et al. 2020. J Bone Miner Res. 35:1352
Ultra-LEAF™ purified rat IgG1k isotype control (RTK2071) BioLegend 400432. Ref to the publication: Yamamoto A, et al. 2021. FASEB J. 35:e21158.
Ultra-LEAF™ purified anti-mouse CD3 (145-2C11) BioLegend 100340. Ref to the publication: Dong MB, et al. 2020. Cell. 178(5):1189-1204.e23
Ultra-LEAF™ purified anti-mouse CD28 (37.51) BioLegend 102116. Ref to the publication: Dong MB, et al. 2020. Cell. 178(5):1189-1204.e23

FITC rat anti-mouse CCR2 (SA203G11) BioLegend 150608. Ref to the publication: Rosina M, et al. 2022. Cell Metab. 34:533
Goat polyclonal anti-mouse CD206 Santa Cruz Biotechnology sc-34577. Ref to the publication: PMID: 27048218
Donkey anti-goat IgG secondary antibody Alexa555™ ThermoFisher scientific A21432. Ref to the publication: DOI: 10.1172/jci.insight.155900
BV605 anti-mouse TCRb (H57-597) BioLegend 109241. Ref to the publication: Delacher M, et al. 2021. Immunity. 54(4):702-720.e17
APC anti-mouse CD3 (17A2) BioLegend 100236. Ref to the publication: Calabrese DR, et al. 2020. J Clin Invest.
APC-Cy7 anti-mouse CD4 (RAM4-5) BioLegend 100526. Ref to the publication: Ma F, et al. 2020. Cell Death Dis. 1.172222222.
BV650 anti-mouse CD8a (53-6.7) BioLegend 100742. Ref to the publication: Flamar AL, et al. 2020. Immunity. 52(4):606-619.e6
PE/Cy5 anti-mouse CD69 (H1.2F3) BioLegend 104509. Ref to the publication: Baptista AP et al. 2019. Immunity. 50(5):1188-1201
PE-CF594 anti-mouse TCRgd (GL3) BD Bioscience 563532. Ref to the publication: Goodman T, et al. Immunogenetics. 1992; 35(1):65-68.
V450 anti-mouse CD11b (M1/70) BD Bioscience 560456. Ref to the publication: Kishimoto TK, et al. Science. 1989; 245(4923):1238-1241
BV785 anti-mouse Ly6C (HK1.1) BioLegend 128041. Ref to the publication: Esmaili S, et al. 2021. Cell Systems. 12(5):432-445.e7.
BV711 anti-mouse F4/80 (BM8) BioLegend 123147. Ref to the publication: Gonalves S, et al. 2021. Cell Reports. 34(11):108860.
PE-Cy7 anti-mouse Tim-4 (RMT-54) BioLegend 130009. Ref to the publication: Daemen S, et al. 2021. Cell Reports. 34(2):108626
PE/Dazzle 594 anti-mouse CD19 (6D5) BioLegend 115553. Ref to the publication: Lee H, et al. 2020. Cell Metabolism. 31(4):822-836
PerCP/CY5.5 anti-mouse Ly6G (IA8) BD Bioscience 560602. Ref to the publication: Fleming TJ, et al. J Immunol. 1993; 151(5):2399-2408.
PE-Cy5 anti-mouse CD11c (N418) BioLegend 117316. Ref to the publication: Uchida A, et al. 2021. J Inflamm Res. 14:3089
AF700 anti-mouse CD45 (30-F11) BD Bioscience 560510. Ref to the publication: Lagasse E, et al. Nat Med. 2000; 6(11):1212-1213
PerCP/CY5.5 anti-mouse NK1.1 (PK136) BioLegend 108727. Ref to the publication: Kobayashi T, et al. 2019. Cell. 176:982
BV650 anti-mouse CCR2 (SA203G11) BioLegend 150613. Ref to the publication: Lee DH, et al. 2019. PLoS One. 14:e0215727.
PE anti-mouse MR1 (26.5) BioLegend 361106. Ref to the publication: McSharry BP, et al. 2020. Cell Reports. 30(9):2948-2962.
eFluor 506 Fixable Viability Dye eBiosciences 65-0866-14. Ref to the publication: Kurtulus S, et al. J Clin Invest. 2015; 125(11), 4053-62
PE/Cy7 anti-mouse TNF-a (MP6-XT22) BioLegend 506324. Ref to the publication: Klarquist J, et al. 2021. Cell Rep. 36:109591.
PE anti-mouse IL-17A (TC11-19H10.1) BioLegend 506904. Ref to the publication: Kimura S, et al. 2020. Nat Commun. 0.620833333.
APC-conjugated anti-human/mouse 5-OP-RU loaded MR1-tetramer. MR1 tetramers were generated by A.Corbett and J.McCluskey and the NIH facility

Animals and other research organisms

Policy information about studies involving animals; ARRIVE guidelines recommended for reporting animal research, and Sex and Gender in Research

Laboratory animals	B6-MAIT CAST and MR1-/- mice were obtained from Olivier Lantz, and both strains have been backcrossed on a C57BL/6J background for more than 10 generations. C57BL6/J mice were ordered in Janvier Labs. Liver fibrosis was induced in 10 to 12-week-old male mice as noticed in the Methods of the manuscript. Mice were then bred in our specific pathogen-free animal facility (SPF), with temperature: 21°C and humidity: 50%. The dark/light cycle was 12h/12h.
Wild animals	No wild animals were used in the study
Reporting on sex	Males were used for animal studies since it has been extensively reported that they are more susceptible to develop liver fibrosis than females (https://doi.org/10.1152/ajpendo.00427.2019). For in vitro experiments in mice, both females and males samples were included. However, low sample size did not enable to draw meaningful conclusions regarding sex and gender differences.
Field-collected samples	No field collected samples were used in the study
Ethics oversight	Experiments were performed in accordance with protocols approved by the Ministère de l'Enseignement Supérieur, de la Recherche et de l'Innovation (APAFIS #30284-2021030912208867), ethic committee APAFIS n°121 Paris Nord

Note that full information on the approval of the study protocol must also be provided in the manuscript.

Flow Cytometry

Plots

Confirm that:

☒ The axis labels state the marker and fluorochrome used (e.g. CD4-FITC).

☒ The axis scales are clearly visible. Include numbers along axes only for bottom left plot of group (a 'group' is an analysis of identical markers).

☒ All plots are contour plots with outliers or pseudocolor plots.

☒ A numerical value for number of cells or percentage (with statistics) is provided.

Methodology

Sample preparation	All the cells analyzed are intrahepatic leukocytes from mice. Following perfusion of 1X PBS through the inferior vena cava, livers were placed into DMEM Dulbecco's Modified Eagle Medium (DMEM, ThermoFisher, Table S3) and digested using the Liver Dissociation kit (Miltenyi, 130-105-807), according to the manufacturer's instructions. Digests were passed through 100µm cell strainer and washed in 30ml DMEM and centrifuged 300g for 10min. Pellets were resuspended in a 33% Percoll (GE Healthcare) diluted with RPMI complete medium (RPMI 1640 (ThermoFisher) containing 10% FBS) at room-temperature and centrifuged 690g for 20min, with minimum break and accelerator. The pellet, containing non-parenchymal cells, was washed with RPMI complete medium. Red blood cells were lysed using 5ml RBC Lysis buffer (BioLegend, 420301) for 3min and washed with RPMI complete medium. The pelleted cells were resuspended in 2ml FACS buffer (1XPBS supplemented with 2%FBS and 2mM EDTA) and transferred to FACS tubes for staining.
Instrument	Data aquisition was performed using BD-Bioscience Fortessa X20 for flow cytometry analyses, and BD-Bioscience LSR-Fortessa ARIA III and BD FACSMelody cell sorter for cell sorting.
Software	Flow cytometry analyses were performed with the FlowJo analysis software V10.7 (Tree Star)
Cell population abundance	For Ly6C hi and Ly6C low MoMac RNAseq 30.000 cells were sorted with purity >90%<br>For MAIT cell sorting, purity was >85%
Gating strategy	Gating strategies are well described in Figure S2, S4 and S5

☒ Tick this box to confirm that a figure exemplifying the gating strategy is provided in the Supplementary Information.
